# A unifying mechanism for cation effect modulating C1 and C2 productions from CO_2_ electroreduction

**DOI:** 10.1038/s41467-022-33199-8

**Published:** 2022-09-19

**Authors:** Seung-Jae Shin, Hansol Choi, Stefan Ringe, Da Hye Won, Hyung-Suk Oh, Dong Hyun Kim, Taemin Lee, Dae-Hyun Nam, Hyungjun Kim, Chang Hyuck Choi

**Affiliations:** 1 Department of Chemistry, Korea Advanced Institute of Science and Technology, Daejeon, 34141 Republic of Korea; 2 School of Materials Science and Engineering, Gwangju Institute of Science and Technology, Gwangju, 61005 Republic of Korea; 3 Department of Chemistry, Korea University, Seoul, 02841 Republic of Korea; 4 Clean Energy Research Center, Korea Institute of Science and Technology, Seoul, 02792 Republic of Korea; 5 Department of Chemistry, Pohang University of Science and Technology (POSTECH), Pohang, 37673 Republic of Korea; 6 Department of Energy Science and Engineering, Daegu Gyeongbuk Institute of Science and Technology, Daegu, 42988 Republic of Korea

**Keywords:** Electrocatalysis, Electrocatalysis, Computational chemistry

## Abstract

Electrocatalysis, whose reaction venue locates at the catalyst–electrolyte interface, is controlled by the electron transfer across the electric double layer, envisaging a mechanistic link between the electron transfer rate and the electric double layer structure. A fine example is in the CO_2_ reduction reaction, of which rate shows a strong dependence on the alkali metal cation (M^+^) identity, but there is yet to be a unified molecular picture for that. Using quantum-mechanics-based atom-scale simulation, we herein scrutinize the M^+^-coupling capability to possible intermediates, and establish H^+^- and M^+^-associated ET mechanisms for CH_4_ and CO/C_2_H_4_ formations, respectively. These theoretical scenarios are successfully underpinned by Nernstian shifts of polarization curves with the H^+^ or M^+^ concentrations and the first-order kinetics of CO/C_2_H_4_ formation on the electrode surface charge density. Our finding further rationalizes the merit of using Nafion-coated electrode for enhanced C2 production in terms of enhanced surface charge density.

## Abstract (web summary)

CO2 reduction rate shows a strong dependence on alkali metal cation identity but a unified molecular picture for underlying mechanism requires further investigation. Using advanced molecular simulations and experimental kinetic studies, here the authors establish a unified mechanism for cation-coupled electron transfer.

## Introduction

Ever-increasing global energy demand is bringing imbalances in the natural cycles, and critically threatening our sustainability. Towards a technological breakthrough that can restore sustainable cycles by rectifying those imbalances, electrochemical CO_2_ reduction reaction (CO_2_RR) is considered promising for converting CO_2_ into valuable chemicals^1–3^, such as CO, CH_4_, and C_2_H_4_. Coinage-metal electrodes of Ag, Au, and Cu are known to be active towards the CO_2_RR^4–7^, on which intense research efforts have been focused^8–13^, while a full understanding of the CO_2_RR mechanism on these catalysts is still not yet at hand.

The most intriguing question is the mechanistic role of alkali metal cations (M^+^) at the catalyst–electrolyte interface. Opposed to the conventional view that M^+^ would spectate the reaction, many experimental reports have evidenced a noticeable dependence of CO_2_RR activity and selectivity on the M^+^ identity^14–17^, often termed a cation effect. Specifically, the selectivity trend towards the C2 product and the CO production activity trend were found to follow the order of Cs^+^ > Rb^+^ > K^+^ > Na^+^ > Li^+^^14,15^, which clearly indicate a certain mechanistic role of M^+^ at the rate-determining step (RDS).

Supposing the proton-coupled electron transfer (PCET) as the RDS, the origin of the cation effect was ascribed to the variation in the local pH at the interface due to the different pKa values of the buffering M^+^^15^. However, various other experiments, where no clear activity change was observed upon (bulk) pH variations, demonstrated the possibility that the RDS involves no proton transfer^12,18–21^. Meanwhile, Chan group suggested a mechanism for the cation effect, called a field effect^16,17,22,23^: the electric double layer (EDL) field formed across the Helmholtz layer would stabilize the intermediates (*e.g*., *CO_2_) via adsorbate dipole-field interaction, which can be modulated by the degree of M^+^ accumulation at the interface^17,24^. Also, using the scanning electrochemical microscopic technique, the Koper group demonstrated an absence of CO_2_RR activity for CO formation without M^+^, which led them to propose a mechanism based on a M^+^-complexation (or coupling) to the CO_2_^−^ intermediate in conjunction with their ab initio molecular dynamics (AIMD) simulation results^13^. Moreover, cation-dependent interfacial water structure has been exploited to understand the cation effect on the CO_2_RR, which yields different electric field strengths^25,26^, adsorption rate^27^, or surface-dependent solvation structure^28^. There further exist other general discussions on the cation effect to the electrocatalytic activity^29^, *e.g*., site-blocking of reactants on the electrode or surface reconstruction, albeit it has not been directly linked with the cation effect on the CO_2_RR.

Towards the definition of a general scheme, at the molecular level, it is thus utmostly required to establish a unified CO_2_RR mechanism based on a systematic assessment, which can elucidate the cation effect on the activity for the CO formation and selectivity towards C–C coupling for multicarbon products. Herein, we investigate the cation-controlled mechanism by reflecting more practical electrolysis conditions via full-equilibrium simulations and flow-type-electrolyzer experiments with gas diffusion electrodes (GDE). Using a quantum-mechanics-based multiscale simulation, offering an accurate description on the EDL structure at atom-scale^30^, we mapped out the cation-coordinating ability to all possible intermediates and established corresponding reaction energy profiles for CO, CH_4_, and C_2_H_4_ productions. The suggested different nature of RDSs, either a cation-coupled electron transfer (CCET) step for CO and C_2_H_4_ productions or a PCET for CH_4_ production, was corroborated by our experiments widely varying the M^+^ concentrations and identities. Of prime interest is that the cation effect results from a cation-dependent electrode surface charge density (|*σ*|). Our mechanism further successfully accounts for recent empirical but breakthrough findings (*e.g*., ionomer effects)^6,31–35^, and highlights the importance of EDL engineering as the next quest for better CO_2_ electrolysis.

## Results and discussion

### To be, or not to be coordinated by a cation

To identify the chemical role of M^+^ during CO_2_RR, we first investigate atomic details of the catalyst–electrolyte interface using density functional theory in classical explicit solvent (DFT-CES) simulation^36^. This method offers an accurate description of the electrified interface at a balanced computational cost, by mean-field coupling of a quantum mechanical description on the catalyst surface with a molecular dynamics description on the liquid structure of the electrolyte phase^37^. Recent advances in computational simulations enable a direct investigation of the electrode-electrolyte structure, highlighting the importance of the atomic arrangement of EDL constituents (e.g. chemisorbed water, Helmholtz ions, etc.) at the buried nanoscale region, which can affect the catalytic reactions under the actual electrochemical conditions^30,38–44^. Compared with the AIMD simulation, often used for modeling the electrochemical interfaces, the DFT-CES enables to investigate electrolyte phase dynamics with many more atoms over a more extended time-scale; multi-thousands of atoms over a few nanoseconds using the DFT-CES *vs*. multi-hundreds of atoms over a few picoseconds using the AIMD^13,38,40,41,45^. The availability of simulations at full length- and time-scales is critical to unbiasedly confirm the possible coordinating ability of electrolyte constituents to the intermediate species since it can provide the full equilibrium-dynamic structure of the electrolyte phase without the influence of initial conditions. Most importantly, we note that the DFT-CES succeeded in unraveling the atomic origin of the famous camel-shaped behavior of the EDL capacitance, confirming its accuracy in describing the EDL structural details^30^.

**Fig. 1:**
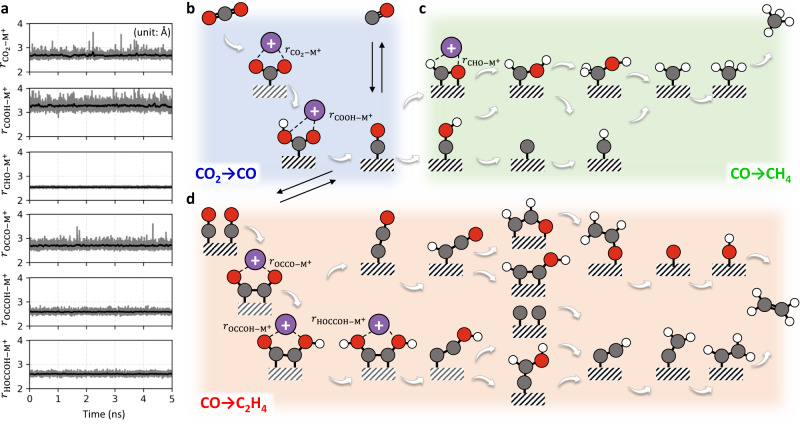
Map of cation-(un)coordinated intermediates during CO_2_RR. **a** Temporal change of distance (*r*) between intermediate and K^+^ during 5-ns DFT-CES trajectory (grey line). Applied potential is −1.0 V_SHE_. The black solid line denotes a moving average of the grey line using a 0.05-ns time window. **b**–**d** Possible intermediates for CO_2_RR, where cation-coordinated species are identified with omitting the water molecules for visual clarity. Reaction paths are denoted using different background colors; blue for CO_2_-to-CO (**b**), green for CO-to-CH_4_ (**c**), and red for CO-to-C_2_H_4_ (**d**). Cation CN to each intermediate is provided in Supplementary Fig. 4, and the species with CN ~ 1 are identified as cation-coordinated intermediates. All cartooned structures are based on the DFT-CES simulation (Supplementary Fig. 1). The dark-grey, red, white, and purple colored ball indicates the C, O, H, and K^+^, respectively, and the electrode is shown with the hatch patterned box.

Using DFT-CES simulations, we investigate the cation-coordinating ability of 28 possible intermediate species that can be formed during the reaction paths of CO_2_RR (Fig. 1 and Supplementary Fig. 1); a path to form CO (Fig. 1b; blue), CH_4_ (Fig. 1c, green), and C_2_H_4_ via a C–C coupling step (Fig. 1d, red). Cu(100) surface, known to be active for C–C coupling reactions^46^, was chosen as the model catalyst surface for CH_4_ and C_2_H_4_ formation paths, as well as Ag(111) surface for CO formation path. At two different potentials of −0.5 V vs. standard hydrogen electrode (SHE) for the potential at point of zero charge (*E*_PZC_) and −1.0 V_SHE_ for the interface charge of −18 μC cm^−2^ (Supplementary Fig. 2), DFT-CES simulations identified 6 intermediate species—*CO_2_, *COOH, *CHO, *OCCO, *OCCOH, and *HOCCOH—to be coordinated by a cation; herein K^+^ (Fig. 1a and Supplementary Figs. 3–5).

**Fig. 2:**
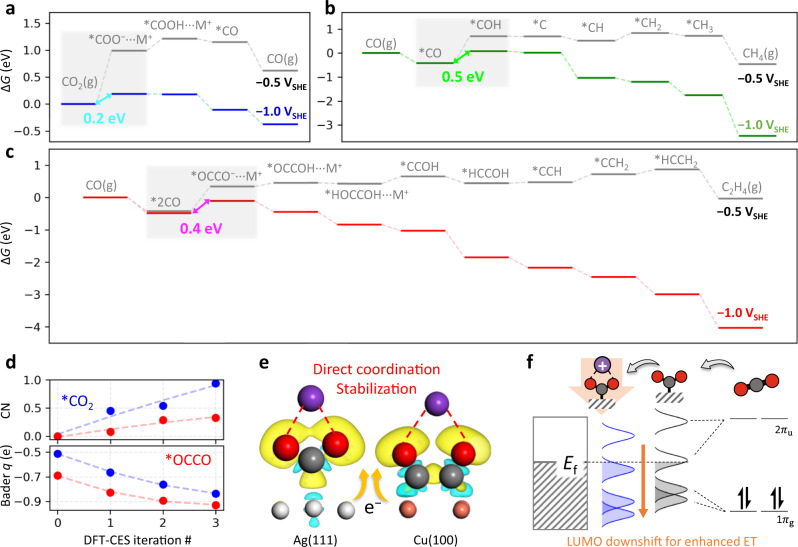
Reaction energy diagrams and cation-coupled electron transfer. **a–c**, Reaction free energy (Δ*G*) diagrams for CO_2_-to-CO on Ag(111) (**a**), CO-to-CH_4_ on Cu(100) (**b**), and CO-to-C_2_H_4_ on Cu(100) (**c**). Applied potentials are −0.5 and −1 V_SHE_. The asterisk (*) indicates the adsorbed state, and the energetically favorable paths, among other possibilities (Supplementary Fig. 6) are selectively shown. The cation-coordinated intermediates are specified by appending ···M^+^. The RDS is denoted by a grey-shaded region. **d**, Change in CN of K^+^ to the *CO_2_ and *OCCO, and that in the Bader charge (Bader *q*) of those two intermediates during DFT-CES iterations at −0.5 V_SHE_. **e**, Cation coupling to the intermediates not only stabilizes them, but also helps an electron transfer to them from the metal electrodes. Isosurface shows a charge difference between the cation-uncoupled case at the 0^th^ iteration step and the cation-coupled case at the last iteration step (isosurface value is 0.0006 e a_0_^−3^). Yellow and cyan colors indicate charge accumulation and depletion, respectively. The water molecules are omitted for visual clarity. **f**, A schematic showing the energy level change of CO_2_, which is based on the PDOS analyses (Supplementary Fig. 9). Before the adsorption, CO_2_ at a linear form has 1π_g_ HOMO state and 2π_u_ LUMO state. When it is adsorbed to the metal, the states are broadened due to the mixing with metal states, and the bent form breaks the degeneracy of 2π_u_ and 1π_g_ states; one level shifts down from the 2π_u_ LUMO state and is partially filled, yielding a radical character. When a cation is coupled to the bent *CO_2_, the LUMO state downshifts further due to the electric field from the cation. This increases the electronic occupation to the *CO_2_, yielding an anionic character of *CO_2_. *E*_f_ denotes the Fermi level.

After constructing reaction paths with explicitly specifying the cation-coordinated intermediate species (as illustrated in Fig. 1b–d), we calculated the reaction free energy profile of each reaction path as shown in Fig. 2a–c (Supplementary Figs. 6 and 7 for all intermediates; see Supplementary Note 1 for the computational details). Full reaction free energy profiles suggest the kinetics of CO, CH_4_, and C_2_H_4_ formations, to be respectively controlled by the RDSs of

1 $$\ast+{{{{{{\rm{CO}}}}}}}_{2}+{{{{{{\rm{M}}}}}}}^{+}+{{{{{{\rm{e}}}}}}}^{-}\to*{{{{{{\rm{COO}}}}}}}^{-}\cdots {{{{{{\rm{M}}}}}}}^{+}$$

2 $$\ast {{{{{\rm{CO}}}}}}+{{{{{{\rm{H}}}}}}}^{+}+{{{{{{\rm{e}}}}}}}^{-}\to*{{{{{\rm{COH}}}}}}$$

3 $$\ast 2{{{{{\rm{CO}}}}}}+{{{{{{\rm{M}}}}}}}^{+}+{{{{{{\rm{e}}}}}}}^{-}\to*{{{{{{\rm{OCCO}}}}}}}^{-}\cdots {{{{{{\rm{M}}}}}}}^{+}$$

Here, reaction (2) is usually termed a PCET, and thus in an analogical sense, reactions (1) and (3) can be termed a CCET.

The proposed RDSs corroborate previous experiments. Previous studies demonstrated a strong dependence of CO formation or C–C coupling rates on the cation identity, *i.e*., cation effect^14–16,47,48^. Also, Monteiro et al. showed a lack of CO_2_RR activity to CO without M^+^, which initiated an intensive discussion about the possibility of CCET^13^. In addition, Chan and coworkers investigated the kinetic importance of proton activity using pH control experiments^12,46^. They found that the pH variation significantly changes the CH_4_ production rate^46^, while the CO and C_2_H_4_ production rates are nearly unchanged on a SHE potential scale^12,46^. The reactions (1) and (3) infer a critical role of M^+^ in the kinetics of CO and C_2_H_4_ formation paths, and reaction (2) shows the kinetic importance of pH in the CH_4_ formation path.

### Nature of cation-coupled electron transfer

Although the CCET is named after the PCET, there is a caveat to understand the nature of CCET in parallel to the PCET. Since cations other than a proton are much heavier than an electron, the nonadiabatic effect can no longer play a role in determining the transfer rate^49^. Rather, it is more reasonable to consider a Born-Oppenheimer-type picture, where an electron is adiabatically transferred to the intermediate species along the reaction coordinate for the cation-coupling.

Analysis of the electronic response of the catalyst–adsorbate system during DFT-CES iterations provides valuable insight on the nature of CCET, which is indeed an adiabatic response of the electron density upon the electrolyte structure change. Figure 2d shows the change of cation coordination number (CN) to the key intermediate species of *CO_2_ and *OCCO, and the change of their partial charges. We find no cation-coupling at the 0^th^ iteration step, but the electron density between the metal and the adsorbate is redistributed during iterations, which enables the cation-coupling (Fig. 2e and Supplementary Fig. 8). Projected density of states (PDOS) shows that the lowest unoccupied molecular orbital (LUMO) of the adsorbate is downshifted after the cation-coupling due to the field generated by the cation (Supplementary Fig. 9). This increases the electronic occupation of LUMO^50^, which partially reduces the adsorbate species (Fig. 2f), yielding the partial charges of *CO_2_ and *OCCO to be −0.7 and −0.9, respectively. The results of DFT-CES simulation are in agreement with the previous novel findings showing that the alkali metal cations can stabilize the *CO_2_ intermediate^13,51^ and *OCCO intermediate^52,53^, respectively.

The entire electron density redistribution, which is associated with the adiabatic reaction coordinate not only for the cation-coupling but for the adsorbate-binding^50^, can be conceptualized in two different pictures; either an electronic polarization or an electron transfer from metal to adsorbate. For the *CO_2_ case, the former concept implies an absence of ET at the RDS;

4 $$\ast+{{{{{{\rm{CO}}}}}}}_{2}+{{{{{{\rm{M}}}}}}}^{+}\to*{{{{{{\rm{CO}}}}}}}_{2}\cdots {{{{{{\rm{M}}}}}}}^{+}$$

which is followed by a subsequent fast ET^50^. Electrostatically, the polarization induces a dipole that can be stabilized by an external field. Thus, the field effect perspective, suggested by Chan group^16,17,22,23^, can be further elaborated by identifying the atomic structural details of the cation that generates the field to stabilize the dipole induced at the metal-intermediate interface.

On the other hand, the latter concept of electron transfer literally implies that the intermediate such as *CO_2_ should be reduced into *CO_2_^−^ at the final stage of the adiabatic reaction path of adsorption and cation-coupling, i.e., the reaction (1) becomes an appropriate expression for the RDS. Although this is similar to what is suggested by Koper group^13^, they illustrated a stepwise path of reductive adsorption and cation-coupling. The ET perspective can be further supported by the intermediate partial charge close to −1, and the PDOS demonstrating an electron-filling to the downshifted LUMO after the cation-coupling. However, strictly speaking, electrons exist as a cloud of indistinguishable quantum-mechanical particles. Consequently, the distinction between polarization and transfer depends on a hypothetical partitioning of the electron density in space, and both are the same phenomenon if there is a significant electronic overlap between the metal and the adsorbate^54^. Thus, there is no fundamental difference between the reaction (4) + fast ET and the reaction (1), but they are two different viewpoints on the same phenomenon; the former stems from more continuum-level and electrostatic perspective, while the latter stems from more atomic-level and charge-transfer perspective.

### Cation concentration-dependent Nernstian shifts

**Fig. 3:**
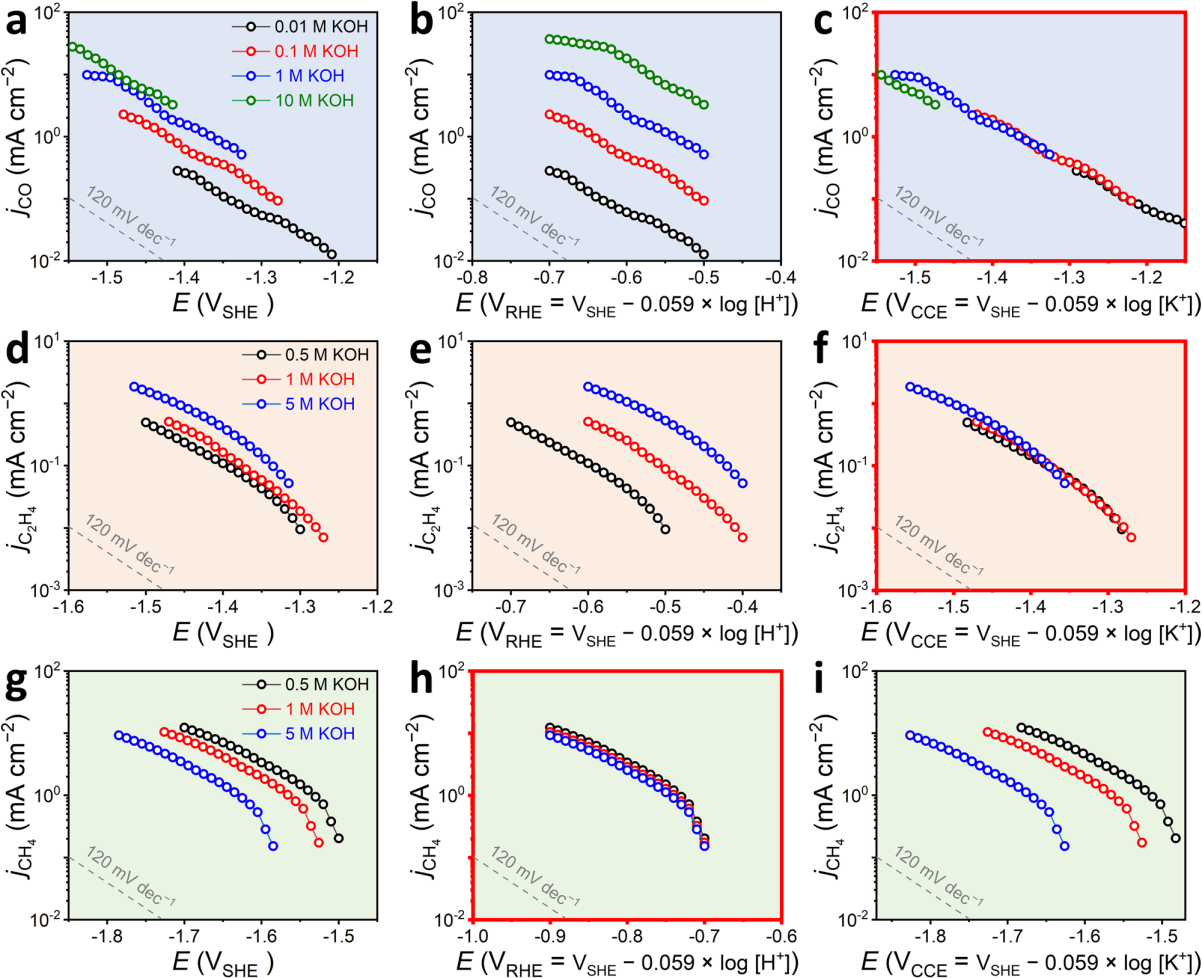
Electrochemical CO_2_RR and CORR in various electrolytes. **a–c** The CO_2_-to-CO conversion was measured on the Ag electrode in 0.01–10 M KOH electrolytes. **d**–**f** The CO-to-C_2_H_4_ and **g**–**i** the CO-to-CH_4_ conversions were measured on the Cu electrode in 0.05–5 M KOH electrolytes. All the electrolysis measurements were performed in an electrochemical flow cell. The polarization curves are plotted with respect to the **a**, **d**, **g** SHE, **b**, **e**, **h** RHE (V_SHE_ – 0.059 × log[H^+^]), or **c**, **f**, **i**, CCE (V_SHE_ – 0.059 × log[K^+^]) scales. The grey lines indicate a Tafel slope (typically plotted as an inverse function of the present polarization curve) of 120 mV dec^−1^. Collapses of the polarization curves are found for the CO and C_2_H_4_ formations upon the CCE scale and for the CH_4_ formation upon RHE scale, which are highlighted with red boxes.

To elucidate the proposed CCET mechanism, we investigated CO_2_-to-CO conversion on the polycrystalline Ag electrode in various concentrations of KOH electrolytes (0.01–10 M) using a flow cell reactor (see Methods, Supplementary Figs. 10 and 11). The CO_2_RR polarization results are provided in Fig. 3a–c (with respect to different reference potential scales; Supplementary Fig. 12 for the Faradaic efficiency (FE)). On both SHE (Fig. 3a) and reversible hydrogen electrode (RHE, Fig. 3b) scales, a partial current density of CO (*j*_CO_) shows considerable deviations in their polarization curves and is promoted as the KOH concentration increases. The departure of *j*_CO_ curves in SHE and RHE scales implies that CO_2_RR kinetics does not simply depend on the electrode potential (i.e., * + CO_2_ + e^−^ → *CO_2_^−^), nor does its RDS accompanies the PCET step (i.e., * + CO_2_ + H^+^ + e^−^ → *COOH), reasonably leading us to account for K^+^-coupled mechanism in their RDSs on the basis of our simulation results.

Hence, we re-plotted the polarization curves with respect to an alkali metal cation concentration-corrected electrode (CCE) scale (Fig. 3c), defined here as *E*_CCE_ = *E*_SHE_ − 0.059 × log[M^+^], where [M^+^] denotes a bulk cation concentration^55^. This plot identifies a collapse of the *j*_CO_ polarization curves independent of the KOH electrolyte concentration, corresponding to a Nernstian potential shift of *ca*. 60 mV per log[K^+^] on the SHE scale. An identical trend was also confirmed in 0.01 M KOH + 0–0.495 M K_2_CO_3_ electrolytes (Supplementary Figs. 13 and 14), in which only the K^+^ concentration was varied but electrolyte pH was almost untouched (Supplementary Fig. 15). With a Tafel slope of 120–130 mV dec^−1^ for the *j*_CO_, the results support that the RDS for CO formation path is the first ET step involving the K^+^-coupling, i.e., the reaction (1). Notably, the collapse of *j*_CO_ curves in the CCE scale is not a singular event that occurs limitedly on Ag electrode in the alkaline electrolyte, but can also be found in other representative electrodes for efficient CO production, e.g., Au and NiNC (Supplementary Figs. 16–19). However, the CO_2_RR in acidic electrolytes is unable to be explained together due to a partial displacement of cations by the protons (or hydronium ions)^56^ (Supplementary Fig. 20).

Afterwards, the C_2_H_4_ formation path, which was also predicted to follow the CCET step, was investigated. Herein, instead of the CO_2_RR, CO reduction reaction (CORR) was chosen as a model reaction for clearer reaction kinetic studies on a polycrystalline Cu electrode (Supplementary Fig. 21**;** see Supplementary Note 2 and Supplementary Figs. 22–25 for CO_2_RR on the Cu electrode). CORR was also performed in various electrolytes having different pHs and K^+^ concentrations, *i.e*., 0.5–5 M KOH (Fig. 3d–i) and 0.5 M KOH with 0.25/2.25 M K_2_CO_3_ (Supplementary Figs. 26 and 27), and the partial current density of ethylene (*j*_C2H4_) was plotted with respect to the SHE, RHE, and CCE scales. As the *j*_CO_ trend, the results exhibited a collapse of the *j*_C2H4_ curves on the CCE scale, but marked departures on the SHE and RHE scales, with a Tafel slope of *ca*. 120 mV dec^−1^ (Fig. 3d–f). Therefore, RDS for the C_2_H_4_ formation of CORR is also identified as the first ET step coupled with one K^+^ transfer, i.e., the reaction (3).

On the other hand, the partial current densities of CH_4_ (*j*_CH4_), measured by CORR on the Cu electrode, are collapsed on the RHE scale, but considerable deviations can be seen on the SHE and CCE scales (Fig. 3g–i). Their Tafel slopes are *ca*. 120 mV dec^−1^, indicating that RDS of the CH_4_ formation from CORR is the first ET step via PCET, i.e., the reaction (2). Therefore, our experimental findings for all CO, C_2_H_4_, and CH_4_ formation paths greatly support the DFT-CES predictions that the two formers accompany the CCET while the latter does the PCET in their RDSs.

### CCET kinetics controlled by surface charge density

Besides the electrolyte pHs and K^+^ concentrations, CO_2_RR activity or selectivity is known to be affected by the M^+^ identity, i.e., a cation effect^14,15^. The M^+^-dependent CO_2_RR activity was also reproduced in our experiments, performed on Ag (Supplementary Fig. 28) and Cu (Supplementary Fig. 24) electrodes. They show activity trend of Cs^+^ > Rb^+^ > K^+^ > Na^+^ > Li^+^ for CO and C_2_H_4_ formations but opposite trend for CH_4_ formation. An identical trend was also found for CORR on Cu electrode (Supplementary Fig. 29).

Cation-dependent activity change could be ascribed to the different intermediate stabilization ability of different M^+^ at the RDS, where the cation is coupled. However, the larger cation has a longer coordination distance, when it develops an inner-sphere interaction with the negatively charged intermediate (*e.g*., *COO^−^ or *OCCO^−^), resulting in a less coulombic stabilization of the intermediate^13^, and thus predicting an activity trend opposite to that of the experiment.

Instead, reaction kinetic study, which can provide definite evidence on reaction mechanism^57^, unravels that different CO or C_2_H_4_ production rates depending on the M^+^ identity (and its bulk concentration) are primarily attributed to different |*σ*|. Considering that the CCET steps of the reactions (1) and (3) govern overall CO and C_2_H_4_ production rates, respectively, the Butler-Volmer kinetics at large cathodic overpotentials yield

5 $${j}_{{{{{{\rm{CO}}}}}}}={n}_{1}F{k}_{1}{P}_{{{{{{{\rm{CO}}}}}}}_{2}}{C}_{{{{{{{\rm{M}}}}}}}^{+}}{e}^{-{\alpha }_{{{{{{\rm{c}}}}}}}F\left({E-{E}^{{0}^{{\prime} }}}_{\left({{{{{\rm{Reaction}}}}}}\;(1)\right)}\right)/{RT}}$$

6 $${j}_{{{{{{\rm{C}}}}}}2{{{{{\rm{H}}}}}}4}={n}_{2}F{k}_{2}{P}_{{{{{{\rm{CO}}}}}}}^{2}{C}_{{{{{{{\rm{M}}}}}}}^{+}}{e}^{-{\alpha }_{{{{{{\rm{c}}}}}}}F\left({E-{E}^{{0}^{{\prime} }}}_{\left({{{{{\rm{Reaction}}}}}}\;(3)\right)}\right)/{RT}}$$

where *F*, *R*, and *T* are the Faraday constant, gas constant, and temperature, respectively. ${n}_{1(2)}$ and ${k}_{1(2)}$ are the number of electrons and rate constant involved in the CO_2_-to-CO (or CO-to-C_2_H_4_) conversion reaction, respectively, and$\,{P}_{{{{{{{\rm{CO}}}}}}}_{2}({{{{{\rm{CO}}}}}})}$ denotes the CO_2_ (CO) partial pressure. ${{E}^{{0}^{{\prime} }}}_{\left({{{{{\rm{Reaction}}}}}}(1)\right)}$ and ${{E}^{{0}^{{\prime} }}}_{\left({{{{{\rm{Reaction}}}}}}(3)\right)}$ are the formal reduction potential of the elementary step reaction (1) and reaction (3), respectively, and ${\alpha }_{{{{{{\rm{c}}}}}}}$ is the cathodic charge transfer coefficient.

According to the equations, the reaction rates are determined by the local cation concentration at the interface, ${C}_{{{{{{{\rm{M}}}}}}}^{+}}$. Unfortunately, this parameter is not straightforwardly measurable or even defined^58,59^. Instead, it can be reasonably hypothesized that the excess cations at the EDL^59^, which locates there to screen the electrode surface charge, will involve in the CCET reaction. If this assumption is true, the CO and C_2_H_4_ production rates should show the first-order reaction kinetics on the |*σ*| at the same ${P}_{{{{{{{\rm{CO}}}}}}}_{2}({{{{{\rm{CO}}}}}})}$ and *E* on the SHE scale, because ${C}_{{{{{{{\rm{M}}}}}}}^{+}}$ will be equal to or (at least) proportional to the |*σ*|^59^.

The |*σ*| at a certain potential (${E}^{{\prime} }$) can be estimated by integrating differential capacitance (*C*_diff_) from the *E*_PZC_ (Supplementary Fig. 30), using the following equation^60^.

7 $$|\sigma |=|{\int }_{{E}_{{{{{{\rm{PZC}}}}}}}}^{E{\prime} }{C}_{{{{{{\rm{diff}}}}}}}\,{{{{{\rm{d}}}}}}E|$$

**Fig. 4:**
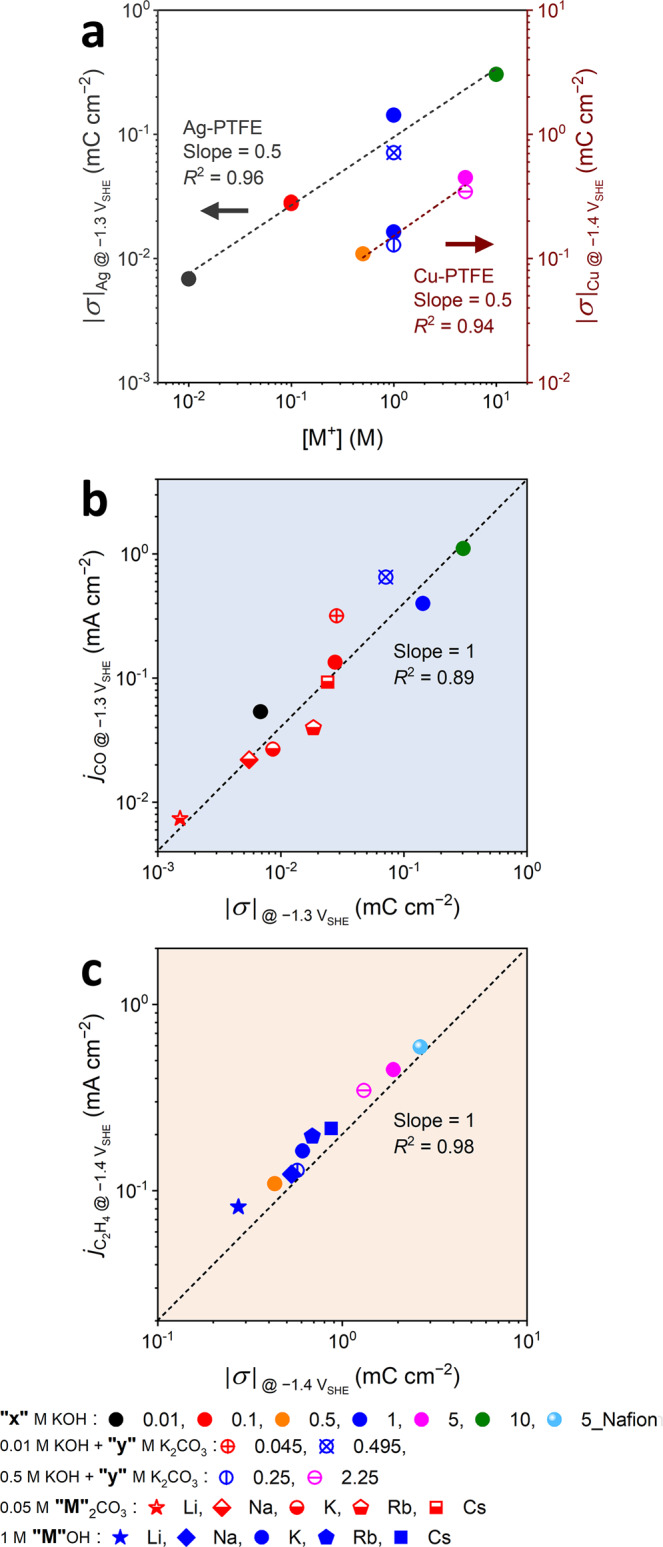
Reaction kinetic studies. **a** Correlation plots between [M^+^] and |*σ*| for the Ag and Cu electrodes. Correlation plots of **b** *j*_CO_ on the Ag electrode and **c**, *j*_C2H4_ on the Cu electrode with |*σ*|. The data was collected at −1.3 V_SHE_ for the Ag electrode (CO_2_-to-CO formation) and −1.4 V_SHE_ for the Cu electrode (CO-to-C_2_H_4_ formation). Electrolytes used for gathering these data can be classified into five different categories: KOH electrolytes with different concentrations (filled circles), 0.01 M KOH electrolytes with additional K_2_CO_3_ salt (double-crossed circles), 0.5 M KOH electrolytes with additional K_2_CO_3_ salt (single-crossed circles), 0.05 M M_2_CO_3_ electrolytes (half-filled symbols), and 1 M MOH electrolytes (filled symbols), where M is Li, Na, K, Rb, and Cs. In addition, a result collected on the Nafion-coated Cu electrode in 5 M KOH electrolyte (a filled circle with pale blue color) was also provided. Here, the total M^+^ concentration and its identity are distinguished by color and symbol shape, respectively. Guidelines for the slopes are indicated by a dashed line.

The staircase potentio electrochemical impedance spectroscopy (SPEIS) reveals magnified *C*_diff_ values as [M^+^] increases or cation size becomes larger (Supplementary Figs. 31 and 32), consequently leading to a wide |*σ*| range, 0.001–0.304 mC cm^−2^ at −1.3 V_SHE_ for Ag electrode and 0.082–0.447 mC cm^−2^ at −1.4 V_SHE_ for Cu electrode. A correlation between |*σ*| and [M^+^] identifies that |*σ*| is proportional to [M^+^]^0.5^ (Fig. 4a), which agrees with the simple prediction using the Gouy-Chapman theory^61^. Notably, their relationship greatly rationalizes the collapse of kinetically described *j*_CO_ or *j*_C2H4_ upon thermodynamically (or Nernstianly) M^+^ concentration-corrected potential (*i.e*., CCE) scale as shown in Fig. 3 (see Supplementary Note 3 for detailed discussion), inferring their RDS to be involved with the CCET path.

More evidently, both a correlation plot between *j*_CO_ and |*σ*| at −1.3 V_SHE_ and that between *j*_C2H4_ and |*σ*| at −1.4 V_SHE_ show a slope of unity in the logarithmic scale (Fig. 4b, c). The first-order kinetics of *j*_CO_ and *j*_C2H4_ on |*σ*| clearly demonstrates our mechanism again that their RDSs accompany the CCET step, i.e., reactions (1) and (3). Considering that data gathered with various M^+^ identities locates on the linear correlation curve of *j*_CO/C2H4_ and |*σ*|, which was plotted from all other control experiments, the changes in |*σ*| mostly ascribes the changes in both *j*_CO_ and *j*_C2H4_. This enables us to conclude that the cation effect on CO and C_2_H_4_ formations to be controlled by the |*σ*|. However, the atomic origin of its cation-species dependence needs to be further unraveled; cation-specific interfacial solvation could play a significant role^13,25–29,51^, suggesting a future research direction.

### Tuning the surface charge density for enhanced C–C coupling

On the basis of above understandings, it can now be rationalized why great C_2_H_4_ productions have been exclusively reported so far with highly concentrated MOH electrolytes (>1 M)^6,31,34^. At the same potential on the SHE scale, high pH electrolyte is not only beneficial for suppressing proton activity and consequent PCET pathways (*e.g*., methane and H_2_ formation), but also induces high |*σ*|, which is indispensable for stabilizing *OCCO^−^ intermediate and thereby lowering energy cost for the C–C coupling step.

**Fig. 5:**
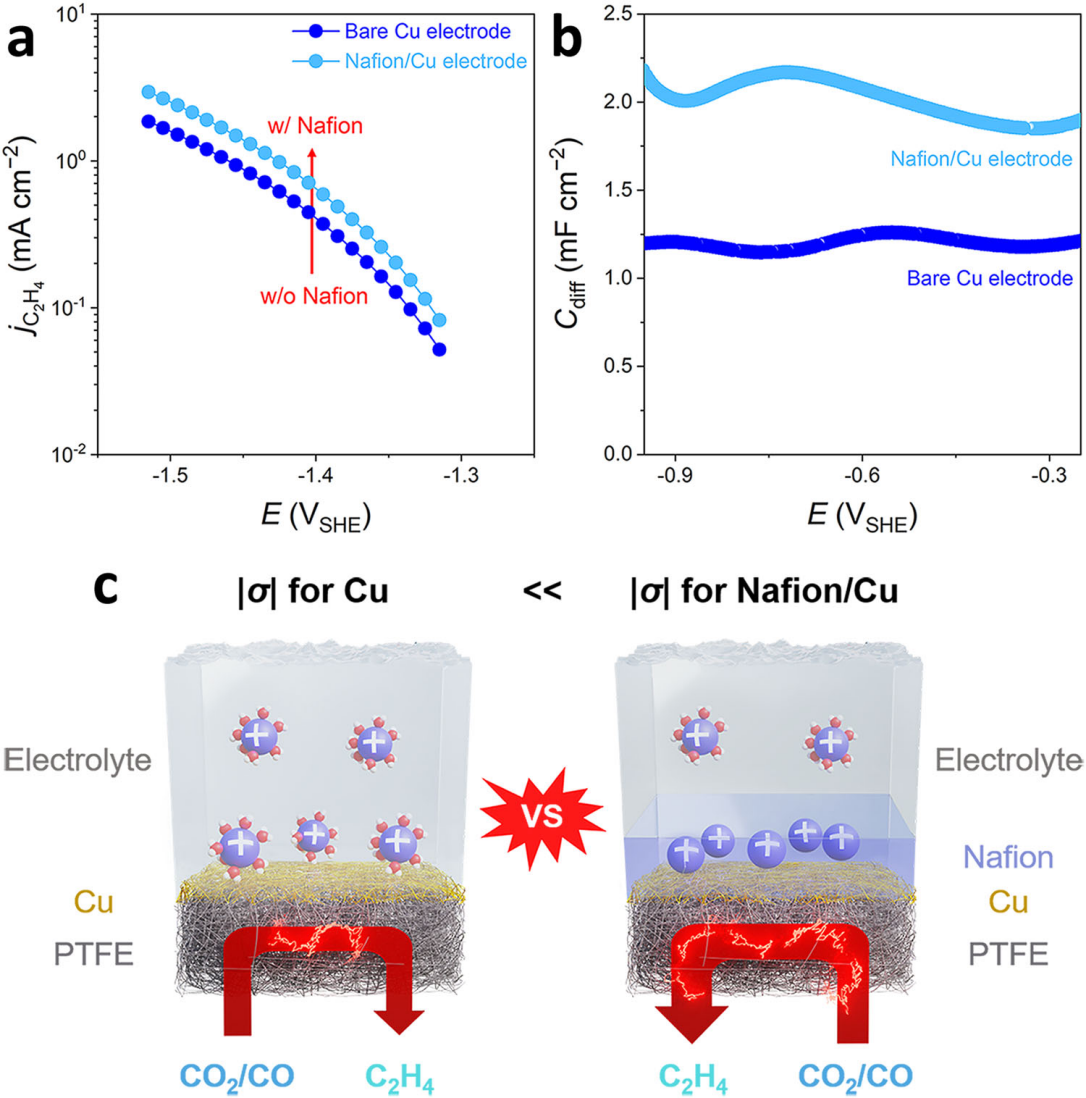
Boosted C_2_H_4_ formation on the Nafion-coated Cu electrode. **a** The *j*_C2H4_ *vs*. potential curves and **b** the *C*_diff_ curves, measured on bare and Nafion-coated Cu electrodes in 5 M KOH electrolyte. **c** Schematic descriptions of the catalyst–electrolyte interface for the Nafion-free and Nafion-coated Cu electrodes, showing a higher |*σ*| on the Nafion-coated one.

More interestingly, we can further provide a clue to a fundamental origin of modulated CO_2_RR activity and selectivity in the presence of ionomer at the interface, highlighted very recently with boosted C_2_H_4_ and other C2 product formations on the Nafion-coated electrode^6,31–35^. In literature, these empirical findings have been deemed as a result from either high local pH (induced by accumulation of OH^−^ ions at the electrode**–**ionomer interface) and consequent high CO_2_ concentration or better mass transport of ionic species. Similarly, we also found 1.6 times higher *j*_C2H4_ (0.59 mA cm^−2^ at −1.4 V_SHE_) on Nafion-coated Cu electrode during CORR (and CO_2_RR, Supplementary Fig. 33) in a 5 M KOH electrolyte than that on bare Cu electrode (Fig. 5a and Supplementary Fig. 34). Also, as shown in Fig. 5b, their SPEIS results verified a significantly tuned *C*_diff_ value (*~*2 mF cm^−2^) on the Nafion-coated electrode, which was *ca*. 1.6 times larger than that on the bare electrode (*~*1.2 mF cm^−2^). Surprisingly, the *j*_C2H4_ and estimated |*σ*| values for the Nafion-coated electrode lie exactly on a previous trend line in Fig. 4c made upon the bare Cu electrode, unveiling that fundamental origin of the boosted C_2_H_4_ production with Nafion ionomer is due to a higher |*σ*| and consequent promotion of the reaction (3) via the CCET pathway (Fig. 5c).

In summary, we present a CCET-based mechanism for CO_2_RR that identifies the role of cations in modulating the activity and selectivity during CO_2_RR towards CO, CH_4_, and C_2_H_4_ formations. Atomic- and electronic-level elucidation on the catalyst–electrolyte interfacial region, empowered by DFT-CES, helps our understanding on the nature of CCET, corroborating previous experimental findings, and mechanistic suggestions. In addition, we demonstrate distinct Nernstian shifts depending on the bulk cation concentration, and first-order kinetics on the electrode surface charge density, both of which evidence our CCET-based mechanism. Most interestingly, our kinetic study finds the cation effect results from the cation-dependent electrode surface charge density. This understanding not only accommodates past and present efforts to tune the electrochemical interfaces for improved CO_2_RR electrolysis (e.g., high pH operation and ionomer-coating) but also brings up a strategic discussion to maximize the electrode surface charge density for further improvements.

## Methods

### DFT-CES simulations

DFT-CES is a grid-based mean-field theory for the quantum mechanics/molecular mechanics (QM/MM) multiscale simulations^36^, where the interfacial interaction is developed based on QM energetics. This is implemented in our in-house code that combines the Quantum ESPRESSO density functional theory simulation engine and LAMMPS molecular dynamics simulation engine^62,63^. Full computational details can be found in Supplementary Note 1.

### Electrode preparations

The electrodes were fabricated by an e-beam evaporator (Ulvac Inc.; deposition rate = 3 Å s^−1^) for Ag and Au and by a sputter (Ulvac Inc.; deposition rate = 6 Å s^−1^) for Cu at a vacuum level of 10^−6^–10^−7^ Torr. Ag (99.99%), Au (99.99%), and Cu (99.99%) targets were deposited onto polytetrafluoroethylene (PTFE) membrane as a GDE with a pore size of 450 nm, and used as a cathode for CO_2_ and/or CO electrolysis. Their successful preparations were investigated by the X-ray diffraction (XRD; MiniFlex 600, Rigaku) and scanning electron microscope (SEM; SU8230, Hitachi). The XRD pattern was obtained at a 40 kV accelerating voltage and a 15 mA current with a scan rate of 1° min^−1^. The SEM and energy-dispersive X-ray spectroscopy (EDS) were taken at an acceleration voltage of 15.0 kV. The Nafion-coated Cu electrode was prepared by spraying a Nafion ink onto the Cu-PTFE electrode. The Nafion ink was prepared by mixing 2.5 mL of isopropanol and 30 μL of Nafion (5 wt%) solution, and its loading amount was set to 12.5 μg cm^−2^.

NiNC catalyst was prepared from Ni^II^ acetate tetrahydrate (98%, Sigma-Aldrich), phen (≥99%, Sigma-Aldrich), and ZIF-8 (ZnN_4_C_8_H_12_, Basolite Z1200 from Sigma-Aldrich). The precursor mixture (1 g), containing 0.5 wt% Ni with a mass ratio phen/ZIF-8 of 20/80, was homogenized by dry ball-milling (FRITSCH Pulverisette 7 Premium) for 4 cycles of 30 min at 400 rpm, and then pyrolyzed at 1050 °C in Ar (5 N, Daedeok) for 1 h. A ZrO_2_ crucible with 100 zirconium oxide balls of 5 mm diameter was used for the ball-milling procedure. Atomically dispersed Ni sites stabilized upon N-doped carbon were characterized by the X-ray photoelectron spectroscopy (XPS), the extended X-ray absorption fine structure (EXAFS), and the high-angle annular dark-field scanning transmission electron microscopy (HAADF-STEM, FEI; Titan^TM^ 80–300 TEM) equipped with a fast CCD camera (Gatan, Oneview 1095). The XPS signal was collected with a Sigma Probe (Thermo VG Scientific) equipped with a micro-focused monochromator X-ray source. The EXAFS was collected in transmission mode at Pohang Accelerator Laboratory (7D-XAFS beamline) with an energy scale calibration by Ni foil. The NiNC electrode was prepared by spraying NiNC ink—4 mg catalyst, 200 μL Nafion solution (5 wt%), and 2800 μL IPA—onto a carbon paper (1 × 1 cm^2^ active area; TGP-H-090 with a 20 wt% PTFE content Toray) with a 1 mg cm^−2^ NiNC. Prior to the NiNC electrode fabrication, hydrophobic mesoporous layer (MPL, preventing electrolyte leakage) was additionally introduced on the carbon paper by spraying a mixture of 100 mg Ketjen black EC-300J, 100 mg PTFE (60 wt%, Sigma-Aldrich), and 20 mL IPA (99.5%, Sigma-Aldrich) with 2 mg cm^−2^ Ketjen black EC-300J loading, and subsequently by heat-treatments at 513 and 613 K under N_2_ atmosphere for 30 min each.

### Electrochemical investigations

All electrochemical measurements were performed with a VMP-300 potentiostat (Bio-Logic). The CO_2_ (4 N, Daedeok) and CO (4 N, Samjung) were electrolyzed in a home-made electrochemical flow cell (Supplementary Fig. 10)^6,64^, in which a working electrode and a saturated Ag/AgCl reference electrode (RE-16, EC-Frontier) were physically separated from a Ni-foam counter electrode (MTI Korea) by an anion exchange membrane (AEM; fab-pk-130, Fumasep). Electrolytes were prepared using deionized water (≥18.2 MΩ, Arium® mini, Sartorius) with various chemicals (all supplied from Sigma-Aldrich): KOH (99.99%), K_2_CO_3_ (99.995%), Li_2_CO_3_ (99.999%), Na_2_CO_3_ (99.95–100.05%), Rb_2_CO_3_ (99.8%), Cs_2_CO_3_ (99.995%), LiOH (98%), NaOH (≥98%), RbOH (99.9%), CsOH (99.95%), HClO_4_ (70 %,), KH_2_PO_4_ (99.0%), H_3_PO_4_ (85 wt%), KHCO_3_ (99.95%), and NaF (>99%). The electrolytes continuously flowed into both anode and cathode compartments of the electrochemical cell with a flow rate of 5 mL min^−1^. In the cathode compartment, CO_2_ or CO gas flowed at the back of the working electrode at a flow rate of 20 mL min^−1^. The reference electrode was calibrated against a Pt wire electrode (CE-1, EC-Frontier) in H_2_-saturated electrolytes and converted to the RHE scale before every single measurement. The SHE and CCE scales were estimated by *E*_SHE_ = *E*_Ag/AgCl_ + 0.197 V and *E*_CCE_ = *E*_SHE_ – 0.059 × log[M^+^], respectively. The CO_2_RR and CORR were conducted by a chronoamperometry (CA) for 1 h at each potential, and their polarization curves were manually IR-corrected (MIR, 85%). All gas products were analyzed using an online gas chromatograph (YL6500 GC, YL Instrument) equipped with a thermal conductivity detector (TCD) and a flame ionization detector (FID). A Carboxen-1000 column (12390-U, Supelco) was used for both TCD and FID, and Ar (99.999%) was used as a reference gas.

The *C*_diff_ of Ag and Cu electrodes were measured using a conventional three-electrode system. A polycrystalline Ag (99.998%, Alfa Aesar) and Cu foils (99.99+%, Goodfellow), a graphite rod, and a saturated Ag/AgCl electrode were employed as the working, counter, and reference electrodes, respectively. Prior to every single measurement, the Ag electrode was chemically polished using the following procedure. The Ag electrode was first immersed in a solution mixture of 0.3 M KCN (≥96%, Sigma-Aldrich) and H_2_O_2_ (29–32%, Alfa Aesar) with a volume ratio of 1.5:1 for 3 s, during which gas was vigorously evolved, and thereafter it was exposed to air for another 3 s. The Ag electrode was subsequently soaked in a 0.55 M KCN solution until the gas evolution ceased, and it was thoroughly washed with DI water. After repeating the chemical polishing procedure 10 times, a highly reflective surface was obtained. For the Cu electrode, it was polished mechanically with alumina slurry (1 and 0.05 µm, R&B Inc.) to remove native Cu oxide. The surface of the polished electrodes was protected by ultrapure water before it was transferred to the electrochemical cell. For measuring the *C*_diff_ of Nafion-coated Cu, 5 wt% Nafion solution was drop-casted onto the Cu foil electrode with a target loading of 12.5 μg cm^−2^, identical to CO_2_ and CO electrolysis studies. The *C*_diff_ was measured by SPEIS with a frequency of 20 Hz and a potential amplitude of 10 mV. The obtained impedance data were fitted by the RC circuit given as *Z* = *R* + 1/*iωC*_diff_ (Supplementary Fig. 35)^17^, where *R* is the solution resistance, and *ω* is the circular frequency. The Ohmic loss was compensated during the SPEIS experiments. The *E*_PZC_ was separately measured in a highly diluted 2 mM NaF solution and was defined as the potential where the smallest *C*_diff_ value was observed.

## Supplementary information

Supplementary information

Peer review file

## Data Availability

All data is available in the main text or Supplementary Information. The main DFT data are available in the ioChem-BD^65^ at 10.19061/iochem-bd-6-162. The main MD data and experimental data are available in the Zenodo at https://zenodo.org/badge/latestdoi/530912301.

## Code availability

The DFT-CES code has been deposited in the Github database without accession code at https://zenodo.org/badge/latestdoi/531050106^66^.
