# Supplementary material for: A unifying mechanism for cation effect modulating C1 and C2 productions from CO2 electroreduction
Source: Nat Commun. 2022 Sep 19;13:5482. doi: 10.1038/s41467-022-33199-8 (PMC9485141; doi:10.1038/s41467-022-33199-8)
Supplement: Supplementary file 1 — Supplementary information [file 41467_2022_33199_MOESM1_ESM.pdf]

Supplementary Information for:

A Unifying Mechanism for Cation Effect Modulating C1 and C2 Productions from CO_2 Electroreduction

AUTHORS: Seung-Jae Shin[1,†], Hansol Choi[2,†], Stefan Ringe[3], Da Hye Won[4], Hyung-Suk Oh[4], Dong Hyun Kim[5], Taemin Lee[6], Dae-Hyun Nam[6], Hyungjun Kim[1,*], and Chang Hyuck Choi[5,*]

Affiliations:

[1]Department of Chemistry, Korea Advanced Institute of Science and Technology, Daejeon 34141, Republic of Korea.

[2]School of Materials Science and Engineering, Gwangju Institute of Science and Technology, Gwangju 61005, Republic of Korea.

[3]Department of Chemistry, Korea University, Seoul 02841, Republic of Korea.

[4]Clean Energy Research Center, Korea Institute of Science and Technology, Seoul 02792, Republic of Korea.

[5]Department of Chemistry, Pohang University of Science and Technology (POSTECH), Pohang 37673, Republic of Korea

[6]Department of Energy Science and Engineering, Daegu Gyeongbuk Institute of Science and Technology, Daegu 42988, Republic of Korea.

*Corresponding authors: linus16@kaist.ac.kr (H.K.) and chchoi@postech.ac.kr (C.H.C)

†These authors contributed equally to this work.

This PDF file includes:

Supplementary Note. 1 to 3

Supplementary Figure. 1 to 35

Supplementary References

Supplementary Note 1. Computational details

Mean-field quantum mechanics/molecular mechanics (QM/MM) multiscale simulation, density functional theory in classical explicit solvents (DFT-CES)[1], is implemented in our in-house code that combines the Quantum ESPRESSO[2] plane-wave density functional theory (DFT) simulation engine and LAMMPS[3] molecular dynamics (MD) simulation engine.

The Ag(111) and Cu(100) electrodes were quantum-mechanically modeled using a three-layer slab with a $(2\sqrt{3} \times 4)$rect surface unit cell with the dimensions of 10.15 Å × 11.72 Å, and (3×3) surface unit cell with the dimensions of 10.90 Å × 10.90 Å. The projector-augmented-wave (PAW)[4] method was used with a kinetic energy cutoff of 50 Ry. Gaussian smearing was used with a value of 0.2 eV, and the Perdew–Burke–Ernzerhof (PBE) exchange-correlation functional was employed[5]. A (3 × 3 × 1) Γ-centered k-point grid was used to sample the reciprocal space, and a dipole correction along the z-direction was applied to block the unphysical interaction between the images of the cells. The geometry of every intermediate, except for the $*CO_2$ and *OCCO, was optimized in vacuum, and fixed during the DFT-CES iterations. Because $*CO_2$ and *OCCO are not optimized in vacuum, their geometries were optimized using the implicit solvation method to stabilize the bent and dimerized form, respectively, following the previous report[6]. Bader charge analysis was performed as described in a previous report[7].

The electrolyte phase was modeled using the canonical ensemble MD based on a classical force-field (FF) type description. 1,000 TIP3P-EW[8] water molecules were simulated, and the 0.1 M electrolyte condition was set by adding three K^+ and three bicarbonate ions to the electrolytes. In the case of charged interface with the surface charge density ($|\sigma|$) of -18 μC cm^{-2}, excess electrons were included in the electrode in the QM region, while the 12 excess K^+ were included in the MM region to satisfy the charge neutrality condition. A Nosé–Hoover thermostat[9,10] was used to maintain the temperature at 300 K, with a damping parameter of 100 fs. Periodic boundary conditions were applied along the x- and y-directions, and long-range electrostatic interaction in the simulation cells was treated using the modified particle–particle particle–mesh method for slab geometry[11]. The external potential from the electrode in the QM region was set as follows. The DFT-optimized structure and electrostatic potential obtained from the $(2\sqrt{3} \times 4)$ rect surface unit cell was repeated to fill the

$\left(6\sqrt{3}\times12\right)$rect surface unit cell, resulting in an MD simulation cell dimension of 30.5 Å × 35.2 Å × 50.0 Å for Ag(111) and (3×3) surface unit cell was repeated to fill (9×9) surface unit cell, of which cell dimension for MD simulation makes 32.7 Å × 32.7 Å × 50.0 Å.

The electrolyte components were described based on the FF, and the Lorentz-Berthelot mixing rule was used. TIP3P-EW[8] was used to describe the water molecules, and ion–water interactions were described using parameters presented previously[12]. All adsorbates and bicarbonate ions were modeled using OPLS-AA FF[13]. The van der Waals (vdW) FF parameters between the QM and MM regions were carefully developed to accurately describe the interfacial interactions. To appropriately model the soft exchange repulsion of the metal atom with a diffuse electron density, we used the Buckingham potential with three adjustable parameters: A_{ij}, R_{ij}, and $C_{6,ij}$.

$$U_{ij}^{\mathrm{vdW}}(r) = A_{ij}\exp\left(-\frac{r_{ij}}{R_{ij}}\right) - \frac{C_{6,ij}}{r_{ij}^{6}} \tag{1}$$

To determine the A and R, and C parameters, we obtained the binding energy curves of a single water molecule to the Ag(111) and Cu(100), employing the vdW-corrected DFT method. We employed a vdW-DF2 functional, which is a nonlocal exchange functional that accurately describes the long-range dispersion correlation energy[14]. On the other hand, regarding the ions, to determine the A and R parameters attributed to the Pauli repulsion energy, we obtained binding-energy curves of ions to the Ag hexamer through Hartree-Fock calculations. Because of the existence of a net charge in the system, we avoided using a slab model; instead, we chose the hexamer model with a planar structure with a closed singlet state. It should be noted that the Hartree-Fock energy completely embodies the contribution of the Pauli exchange interaction between closed-shell molecules with no dispersive correlation energy. Therefore, it is ideal to selectively model the Pauli repulsion energy. Hartree–Fock calculations were performed using the NWChem 6.8 software[15] using an effective core potential for the core electrons of the Ag and Cu atom with a def2-TZVP basis set[16]. The process for developing the parameters is shown in **Supplementary Note Fig. 1,** and the parameters are summarized in **Supplementary Note Table 1**. It is of note that the electric double layer (EDL) structure was accurately simulated using current FF parameters, where the characteristic camel-shaped EDL capacitance was successfully reproduced[17].

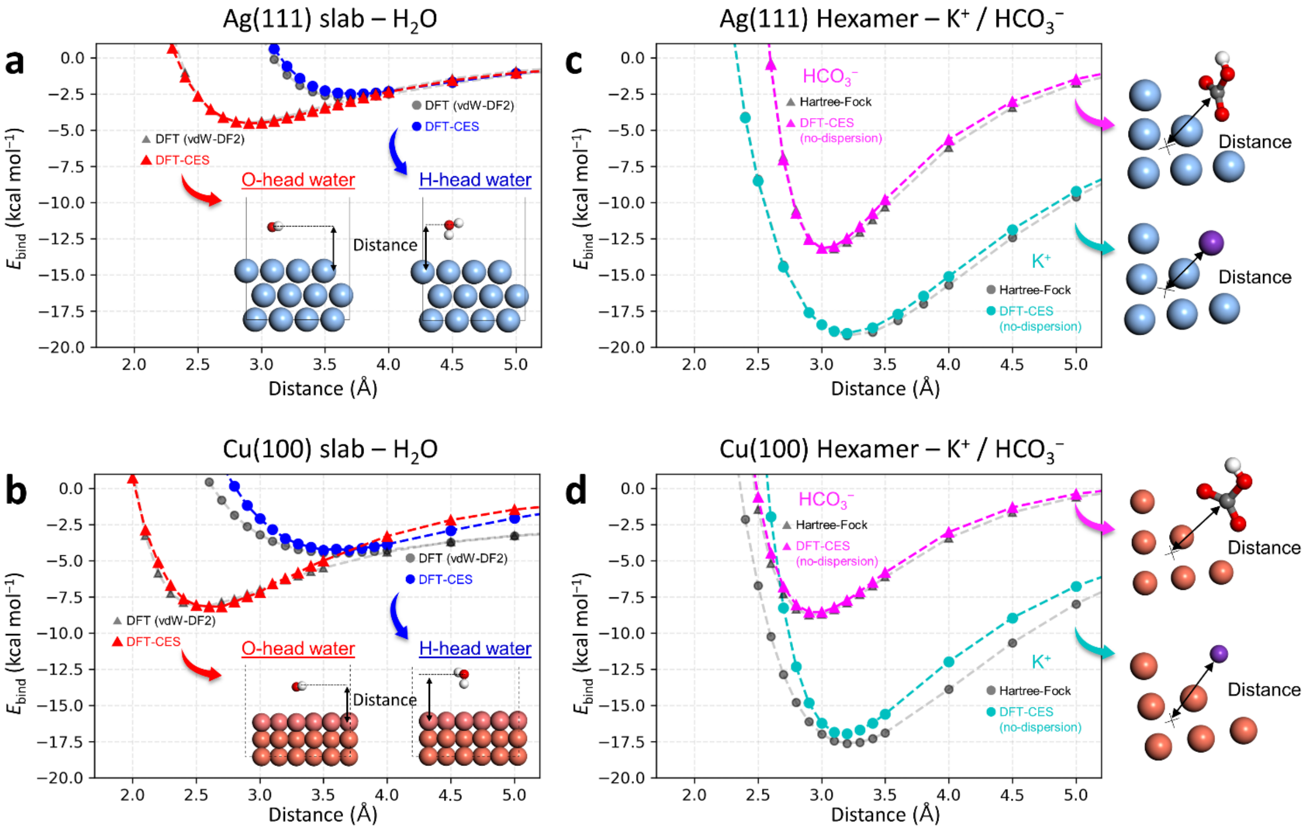

Supplementary Note Figure 1. Optimization of the interfacial vdW parameters for DFT-CES. a–b, The binding energy (E_{bind}) curves of the water molecule and Ag(111) (**a**) and Cu(100) (**b**) were calculated using DFT with vdW-DF2 functional, and they are used to fit the interfacial vdW parameters. For both surfaces, two different configurations for water molecules —H-head and O-head— are considered. **c–d,** E_{bind} curves of the alkali metal cation and bicarbonate anion to the Ag hexamer (**c**) or Cu hexamer (**d**) were calculated using Hartree-Fock theory, which was used to fit the Pauli repulsion parameters of interfacial vdW parameters. The dispersion attraction parameters of C_6 for alkali metal cations and Ag or Cu were determined using real-time time-dependent DFT calculation results[18]. The molecular C_6 parameter of the polyatomic bicarbonate was determined using the quantum mechanical fractionally ionic approach[19], which was assigned to the carbon center. The results are summarized in **Supplementary Note Table 1**.

	A (kcal mol^{-1})	R (Å)	C_6 (kcal mol^{-1} Å^6)
Ag – K^+	10244	0.460	861
Ag – C (HCO_3^-)	2780000	0.240	4253
Ag – H_{water}[17]	3389	0.343	355
Ag – O_{water}[17]	17427	0.348	1739
Cu – K^+	697172	0.271	744
Cu – C (HCO_3^-)	522751	0.267	3688
Cu – H_{water}	4295	0.395	1234
Cu – O_{water}	23354	0.288	701

Supplementary Note Table 1. The vdW parameters between electrolytes and electrodes. Parameters for Ag to H_2O are from the previous study[17].

At every DFT-CES iteration, we performed an MD simulation for 6 ns and sampled the last 5 ns trajectory to calculate the average electrostatic potential of the electrolyte phase, which was used in the subsequent DFT calculation as an external potential. During the DFT calculations, only the electronic structure was optimized based on the external potential obtained from the previous MD iteration. The DFT-CES iteration was repeated until the difference in the DFT total energy between the iterations converged below 0.1 kcal mol^{-1}. In most cases, the total energy converged within three DFT-CES iterations.

The computational hydrogen electrode method is widely used for calculating the energetics of the proton-coupled electron transfer step[20]. The chemical potential of proton and electron is defined as, $\mu[H^+(aq) + e^-] = \mu[H_2(g)] - eU_{SHE} - 0.059 \times pH$, where SHE is the standard hydrogen electrode. In this study, pH was set as 12, which is a consistent value for the experimental condition. U_{SHE} was set as −0.5 V_{SHE}, and −1.0 V_{SHE}, where the detailed procedure to get the electrode potential is shown (**Supplementary Fig. 2**).

Every free energy change (ΔG) is defined as $\Delta G = \Delta E + \Delta E_{ZPE} - T\Delta S$, where ΔE is a reaction energy difference between the product and reactant, which can be directly calculated by DFT, ZPE is zero-point energy, T is the temperature at 298.15 K, and S is entropy. For gas molecules, translational, rotational, and vibrational entropies were considered using particle-in-a-box, rigid rotor, and harmonic oscillator partition functions, respectively. For intermediates adsorbed on the metal, only the vibrational entropy was considered with neglecting the motion of electrode by using the partial Hessian approach[21].

To accommodate a possible DFT error (*e.g.*, a strong self interaction error in the energy of triplet O_2) in predicting the standard reduction potential[22], the reference energies of O_2(g) and CO_2(g) were shifted by 0.44 eV, which bring no qualitative change in the reaction energy diagram. The predicted standard reduction potential values after applying such an *ad-hoc* correction are compared with experimental values in **Supplementary Note Table 2**.

	Exp (V_{SHE})	DFT (V_{SHE})	RMSE (V)
$CO_2(g) + H_2(g) \rightarrow CO(g) + H_2O(l)$	−0.1	−0.1	0.00
$H_2(g) + 1/2O_2(g) \rightarrow H_2O(l)$	1.23	1.23	0.00
$CO(g) + 3H_2 \rightarrow CH_4(g) + H_2O(l)$	0.26	0.29	0.03
$2CO(g) + 4H_2(g) \rightarrow C_2H_4(g) + 2H_2O(l)$	0.17	0.21	0.04

Supplementary Note Table 2. Comparison of the predicted and experimental standard reduction potential values.

Supplementary Note 2. Derivation of a kinetic equation for CO_2-to-C_2H_4 conversion.

Electrochemical CO_2 conversion to C_2H_4 accompanies a series of CO_2 reduction steps, during which the *CO is known as a key intermediate prior to its coupling step[23–25]. Considering our DFT-CES results (and also corroborated by experiments), which show that cation-coupled electron transfer (CCET) mechanisms govern both CO_2 adsorption and C–C coupling steps, electrochemical CO_2-to-C_2H_4 conversion reaction can be written as the following elementary reaction steps.

$* + CO_2 + M^+ + e^- \rightleftarrows *CO_2^- \cdots M^+$ (2) with $E_1^{0'}$, α_1, k_1, k_{-1}

$*CO_2^- \cdots M^+ + H_2O \rightleftarrows *COOH \cdots M^+ + OH^-$ (3) with k_2, k_{-2}

$*COOH \cdots M^+ + e^- \rightleftarrows *CO + M^+ + OH^-$ (4) with $E_3^{0'}$, α_3, k_3, k_{-3}

$2*CO + M^+ + e^- \rightarrow *OCCO^- \cdots M^+$ (5) with $E_4^{0'}$, α_4, k_4, k_{-4}

where $E_x^{0'}$, α_x, k_x, and k_{-x} are the formal reduction potential, the cathodic charge transfer coefficient, and the rate constant for forward and reverse reactions of each reaction step, respectively. M^+ is an alkali metal cation. The coverage of the reaction intermediate belonging to each reaction step is denoted here as θ_x.

θ_1 = Coverage of $*CO_2^- \cdots M^+$ (6)

θ_2 = Coverage of $*COOH \cdots M^+$ (7)

θ_3 = Coverage of *CO (8)

$\theta_4 = 1 - (\theta_1 + \theta_2 + \theta_3)$ (9)

It is of note that, since the (5) is predicted to be a rate-determining step (RDS), coverages of *OCCO$^{-}\cdots$M^{+} and further reduced intermediates were not considered. Then, the θ_4 indicates coverage of vacant catalytic site. The overall reaction kinetics can thus be written as

$$i = k_4\theta_3^2 C_{\mathrm{M}^+}, \qquad \text{where } k_4 = k_4^0 \exp(-\frac{\alpha_4 F}{RT}(E - E_4^{0'})) \tag{10}$$

where i is the kinetic current, k^0 is the standard rate constant, F is the Faraday constant, R is the gas constant, T is the temperature, and E is the applied potential. C_{M^+} is the local concentration at the interface (same for the C_{OH^-} or C_{CO_2} shown below). Considering pre-equilibrium of (2), (3), and (4) prior to the RDS, (5),

$$k_1 C_{\mathrm{CO}_2} C_{\mathrm{M}^+}(1 - \theta_1 - \theta_2 - \theta_3) = k_{-1}\theta_1 \tag{11}$$

$$k_2\theta_1 = k_{-2}\theta_2 C_{\mathrm{OH}^-} \tag{12}$$

$$k_3\theta_2 = k_{-3}\theta_3 C_{\mathrm{M}^+} C_{\mathrm{OH}^-} \tag{13}$$

From the equations (12) and (13), the θ_2 and θ_3 can be expressed as

$$\theta_3 = \frac{k_3}{k_{-3}}\theta_2 \frac{1}{C_{\mathrm{OH}^-}C_{\mathrm{M}^+}} = \frac{K_3}{C_{\mathrm{OH}^-}C_{\mathrm{M}^+}}\theta_2, \qquad \text{where } K_3 = \frac{k_3}{k_{-3}} \tag{14}$$

$$\theta_2 = \frac{k_2}{k_{-2}}\theta_1 \frac{1}{C_{\mathrm{OH}^-}} = \frac{K_2}{C_{\mathrm{OH}^-}}\theta_1, \qquad \text{where } K_2 = \frac{k_2}{k_{-2}} \tag{15}$$

$$\therefore \theta_3 = \frac{K_2}{C_{\mathrm{OH}^-}} \frac{K_3}{C_{\mathrm{OH}^-}C_{\mathrm{M}^+}}\theta_1 = \frac{K_2 K_3}{C_{\mathrm{OH}^-}{}^2 C_{\mathrm{M}^+}}\theta_1 \tag{16}$$

From the equation (11), θ_1 is given by

$$\theta_1 = \frac{k_1}{k_{-1}} C_{\mathrm{CO}_2} C_{\mathrm{M}^+}(1 - \theta_1 - \theta_2 - \theta_3) = K_1 C_{\mathrm{CO}_2} C_{\mathrm{M}^+}(1 - \theta_1 - \theta_2 - \theta_3), \qquad \text{where } K_1 = \frac{k_1}{k_{-1}} \tag{17}$$

By inserting equations (15) and (16) into equation (17), the θ_1 is given by

$$\theta_1 = \frac{K_1 C_{CO_2} C_{OH^-}{}^2 C_{M^+}}{K_1K_2K_3C_{CO_2} + K_1K_2C_{CO_2}C_{OH^-}C_{M^+} + K_1C_{CO_2}C_{OH^-}{}^2C_{M^+} + C_{OH^-}{}^2} \quad (18)$$

and, by using equation (16), the θ_3 can be expressed as

$$\theta_3 = \frac{K_1K_2K_3C_{CO_2}}{K_1K_2K_3C_{CO_2} + K_1K_2C_{CO_2}C_{OH^-}C_{M^+} + K_1C_{CO_2}C_{OH^-}{}^2C_{M^+} + C_{OH^-}{}^2} \quad (19)$$

Therefore, the overall reaction rate, equation (10), becomes

$$i = k_4\theta_3^2 C_{M^+} = k_4\left(\frac{K_1K_2K_3}{K_1K_2K_3C_{CO_2} + K_1K_2C_{CO_2}C_{OH^-}C_{M^+} + K_1C_{CO_2}C_{OH^-}{}^2C_{M^+} + C_{OH^-}{}^2}\right)^2 C_{CO_2}{}^2 C_{M^+} \quad (20)$$

where $\frac{K_1K_2K_3}{K_1K_2K_3C_{CO_2} + K_1K_2C_{CO_2}C_{OH^-}C_{M^+} + K_1C_{CO_2}C_{OH^-}{}^2C_{M^+} + C_{OH^-}{}^2} = \frac{\frac{K_1^0K_2K_3^0}{\exp(f\eta_1)\exp(f\eta_3)}}{\frac{K_1^0K_2K_3^0C_{CO_2}}{\exp(f\eta_1)\exp(f\eta_3)} + \frac{K_1^0K_2C_{CO_2}C_{OH^-}C_{M^+}}{\exp(f\eta_1)} + \frac{K_1^0C_{CO_2}C_{OH^-}{}^2C_{M^+}}{\exp(f\eta_1)} + C_{OH^-}{}^2}$

$$= \frac{K_1^0K_2K_3^0}{K_1^0K_2K_3^0C_{CO_2} + K_1^0K_2C_{CO_2}C_{OH^-}C_{M^+}\exp(f\eta_3) + K_1^0C_{CO_2}C_{OH^-}{}^2C_{M^+}\exp(f\eta_3) + C_{OH^-}{}^2\exp(f\eta_1)\exp(f\eta_3)}$$

Thus,

$$i = \left(\frac{K_1^0K_2K_3^0}{K_1^0K_2K_3^0C_{CO_2} + K_1^0K_2C_{CO_2}C_{OH^-}C_{M^+}\exp(f\eta_3) + K_1^0C_{CO_2}C_{OH^-}{}^2C_{M^+}\exp(f\eta_3) + C_{OH^-}{}^2\exp(f\eta_1)\exp(f\eta_3)}\right)^2 \times k_4^0 C_{CO_2}{}^2 C_{M^+}\exp(-\alpha_4 f\eta_4) \quad (21)$$

where $f = \frac{F}{RT}$, $\eta_x = E - E_x^{0'}$, $K_x = K_x^0\exp(-f\eta_x)$

Finally, in CO_2 reduction reaction (CO_2RR) to C_2H_4, the resulting kinetic equation is not simply proportional to the C_{M^+}, but a very complex function of the C_{M^+} stemming from the pre-factor of "$k_4^0 {C_{\mathrm{CO}_2}}^2 C_{\mathrm{M}^+} \exp(-\alpha_4 f \eta_4)$", which describes an equilibrium of pre-RDS steps. Therefore, dissimilar to CO reduction reaction (CORR) results, CO_2RR polarization curves, measured in varying electrolytes, result in some deviations upon the alkali metal cation concentration-corrected electrode (CCE) scale with relatively poor fitting parameters in correlations of partial current density of ethylene (j_{C2H4}) *vs.* $|\sigma|$, as shown in **Supplementary Figs. 22–25**. Therefore, CORR was mainly investigated and discussed in the present work for clearer elucidation of cation effects on the C–C coupling step.

Supplementary Note 3. Thermodynamics *vs.* Kinetics: Validation of activity comparison upon thermodynamically (Nernstianly) [M^+]-corrected CCE potential scale.

In this work, our DFT-CES results, which predict that RDSs of CO_2RR to both CO and C_2H_4 accompany the CCET mechanism, were experimentally first corroborated with a collapse of their polarization curve upon the CCE scale.

The polarization curves represent kinetics, typically described by the Butler-Volmer equation. On the other hand, the CCE scale is here defined as "$E_{CCE} = E_{SHE} - 0.059 \times \log[M^+]$", basically inferring a compensation of a potential contribution of alkali metal cation concentration according to the Nernst equation, which represents thermodynamics. Although the Bell-Evans-Polanyi principle provides a model describing an intimate relation between the thermodynamics and kinetics[26,27], these two terms are not freely interchangeable and their discussion needs to be separated. Therefore, in this point of view, the collapse of CO_2RR (and CORR) polarization curves upon the CCE scale is interesting, but fundamentally it is unclear whether it strongly supports our argument that RDSs of the CO and C_2H_4 paths involve the CCET step.

In a multi-electron (n) transfer process, $\mathrm{O} + ne^- \rightleftarrows \mathrm{R}$, with an RDS of the first one-electron transfer step, $(\mathrm{O} + \mathrm{e}^- \rightleftarrows \mathrm{R}')$, its current-potential characteristic is given by

$$i = -nFAk^0_{\mathrm{RDS}}\left[C_{\mathrm{O}}(0)\exp\left\{-\frac{\alpha_c F}{RT}\left(E - E^{0'}_{\mathrm{RDS}}\right)\right\} - C_{\mathrm{R}'}(0)\exp\left\{\frac{\alpha_a F}{RT}\left(E - E^{0'}_{\mathrm{RDS}}\right)\right\}\right] \quad (22)$$

where A is the electrode area, k^0_{rds} is the standard rate constant for the RDS step, $C_{\mathrm{O}}(0)$ (and $C_{\mathrm{R}'}(0)$) is the concentration of species O (and R') at the electrode surface, α_{c} (and α_{a}) is the cathodic (and anodic) charge transfer coefficient, and $E^{0'}_{\mathrm{RDS}}$ is the formal potential of the RDS step[28]. At high cathodic overpotential (η), the current-potential characteristic can be rewritten as the following equation.

$$i = -nFAk^0_{\mathrm{RDS}}\left[C_{\mathrm{O}}(0)\exp\left\{-\frac{\alpha_c F}{RT}\left(E - E^{0'}_{\mathrm{RDS}}\right)\right\}\right] \quad (23)$$

For the case of CO_2RR (or CORR), the O at the RDS is one CO_2 molecule (or two CO molecules) and one M^+, and thus, $C_{\mathrm{O}}(0)$ can be expressed as the following equations.

$$C_{\mathrm{O}}(0)_{\mathrm{CO_2RR}} = \mathrm{K}_1 P_{\mathrm{CO_2}} C_{\mathrm{M^+}}(0) \quad (24)$$

$$C_{\mathrm{O}}(0)_{\mathrm{CORR}} = \mathrm{K}_2 P_{\mathrm{CO}}^2 C_{\mathrm{M}^+}(0) \tag{25}$$

where K_1 (K_2) is the equilibrium constant of gas of inputs and gas at the interface, and P_{CO_2} (P_{CO}) is the partial pressure of CO_2 (CO) in gas input. Because the CO_2 (or CO) is neutral species, the CO_2 (or CO) at the interface can be written as $\mathrm{K}_1 P_{\mathrm{CO}_2}$ (or $\mathrm{K}_2 P_{\mathrm{CO}}^2$). On the other hand, because M^+ is non-neutral species, the equilibrium between its bulk and interface is more complicated. Thus, supposing the surface concentration of $C_{\mathrm{M}^+}(0)$ depends on its bulk concentration ($C_{\mathrm{M}^+}^*$) *via* $C_{\mathrm{M}^+}(0)=\beta {C_{\mathrm{M}^+}^*}^{\gamma}$, where β is a proportional coefficient and γ is an exponent, the equation (23) can be written in terms of $C_{\mathrm{M}^+}^*$;

$$i = -nFAk_{\mathrm{RDS}}^0 \left[\mathrm{K}_1 P_{\mathrm{CO}_2} \beta {C_{\mathrm{M}^+}^*}^{\gamma} \exp\left\{-\frac{\alpha_c F}{RT}\left(E - E_{\mathrm{RDS}}^{0'}\right)\right\}\right]$$

$$= -nFAk_{\mathrm{RDS}}^0 \left[\mathrm{K}_1 P_{\mathrm{CO}_2} \beta \exp\left[-\frac{\alpha_c F}{RT}\left\{E - \left(E_{\mathrm{RDS}}^{0'} + \frac{\gamma RT}{\alpha_c F}\ln C_{\mathrm{M}^+}^*\right)\right\}\right]\right]$$

which is further reduced into the following form at $T = 25$ °C;

$$i = -nFAk_{\mathrm{RDS}}^0 \left[\mathrm{K}_1 P_{\mathrm{CO}_2} \beta \exp\left[-\frac{\alpha_c F}{RT}\left\{E - (E_{\mathrm{RDS}}^{0'} + 0.059\frac{\gamma}{\alpha_c}\log C_{\mathrm{M}^+}^*)\right\}\right]\right] \tag{26}$$

Here, the last term of $E_{\mathrm{RDS}}^{0'} + 0.059\frac{\gamma}{\alpha_c}\log C_{\mathrm{M}^+}^*$ is dictated by a shift of formal potential of the RDS step by $0.059\frac{\gamma}{\alpha_c}$ V per $\log C_{\mathrm{M}^+}^*$. For the special case of $\gamma = \alpha_c$, equation (26) further indicates the same kinetics upon Nernstianly shifting the potential. Also, in case of CORR to C_2H_4 path, the prefactor $\mathrm{K}_1 P_{\mathrm{CO}_2}$ is replaced as $\mathrm{K}_2 P_{\mathrm{CO}}^2$.

Usually the α_c is 0.5, and also, the Gouy-Chapman theory predicts the γ to be 0.5.[29] In addition, our experimental results, as shown in **Fig. 4a**, demonstrate the $|\sigma|$ to be proportional to the square root of the bulk ion concentration, inferring $\gamma = 0.5$. Therefore, $\gamma = \alpha_c$ holds (at least) for the CO_2 and CO electrolysis cases, which clearly rationalizes how the thermodynamics and kinetics are interrelated in CO_2RR (and CORR), supporting the validity of our data interpretation upon the CCE scale.

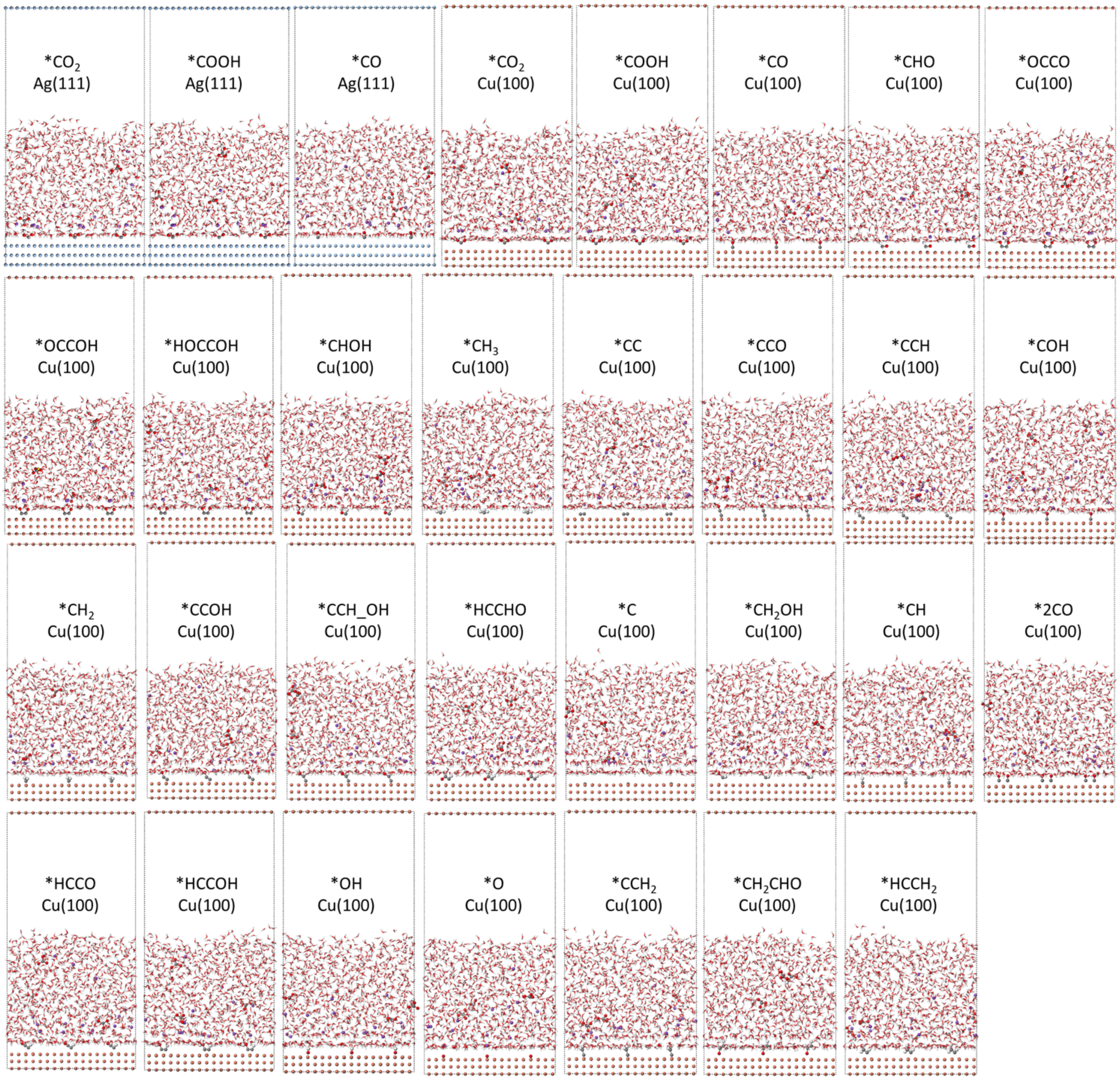

Supplementary Figure 1. Full-scale snapshots of the charged interface with various intermediates from DFT-CES simulations. The representative snapshots of all intermediates on Ag(111) or Cu(100) with 0.1 M $KHCO_3$ electrolyte at −1.0 V_{SHE}. The water molecules are shown as narrow sticks. Purple, dark-grey, red, white, light-blue, and light-orange colors indicate K, C, O, H, Ag, and Cu, respectively.

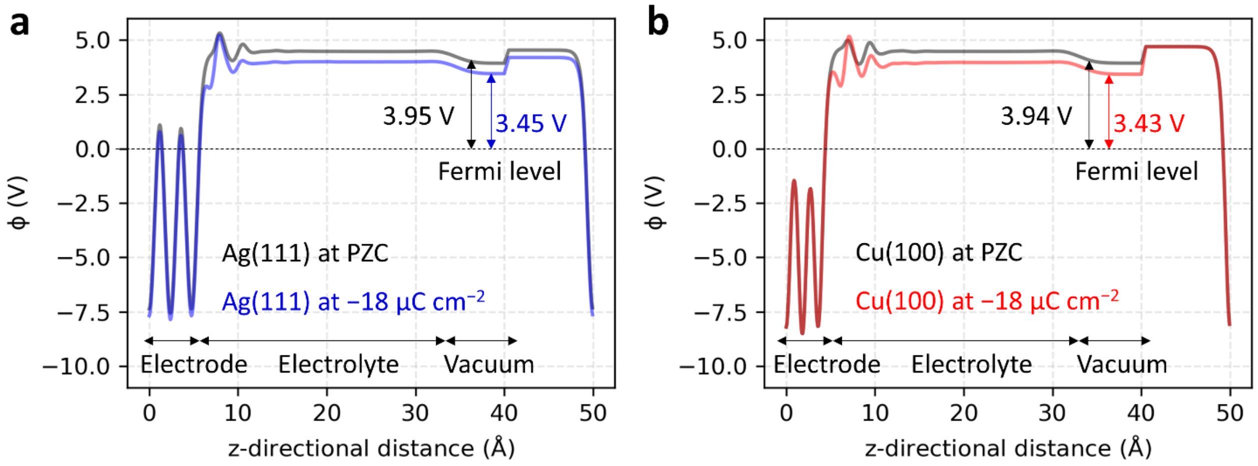

Supplementary Figure 2. Electrostatic potential profiles, ϕ, across the charged interfaces, calculated using DFT-CES. Two different surface charge density (σ) conditions, $\sigma = 0$ for the point of zero charge (PZC), and $\sigma = -18$ μC cm^{-2}, are compared for **a,** Ag(111) electrode and **b,** Cu(100) electrode. The ϕ is aligned to set the Fermi level at $\phi = 0$ V. By subtracting the 4.44 V from the calculated absolute electrode potential values[30], the electrode potential *vs.* standard hydrogen electrode (SHE) are calculated, which are −0.5 V_{SHE} at PZC and −1.0 V_{SHE} at $\sigma = -18$ μC cm^{-2} for both Ag(111) and Cu(100) electrodes.

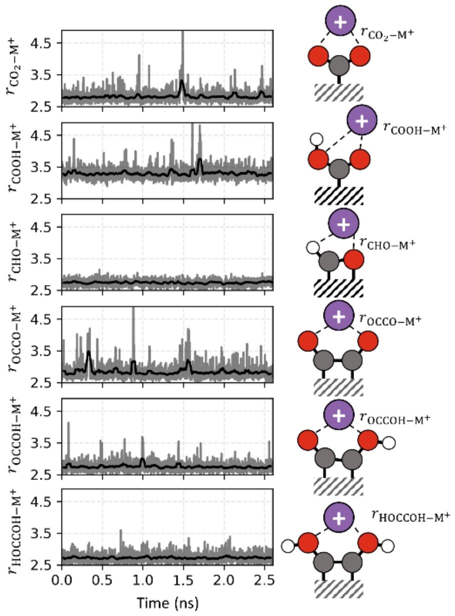

Supplementary Figure 3. Distance between K^+ and cation-coordinated adsorbates. The distance (r) change is plotted as a function of the simulation time in a grey color. The intermediates are adsorbed on the metal electrode at the −0.5 V_{SHE}. The black solid line denotes a moving average of the grey line using a 0.05-ns time window.

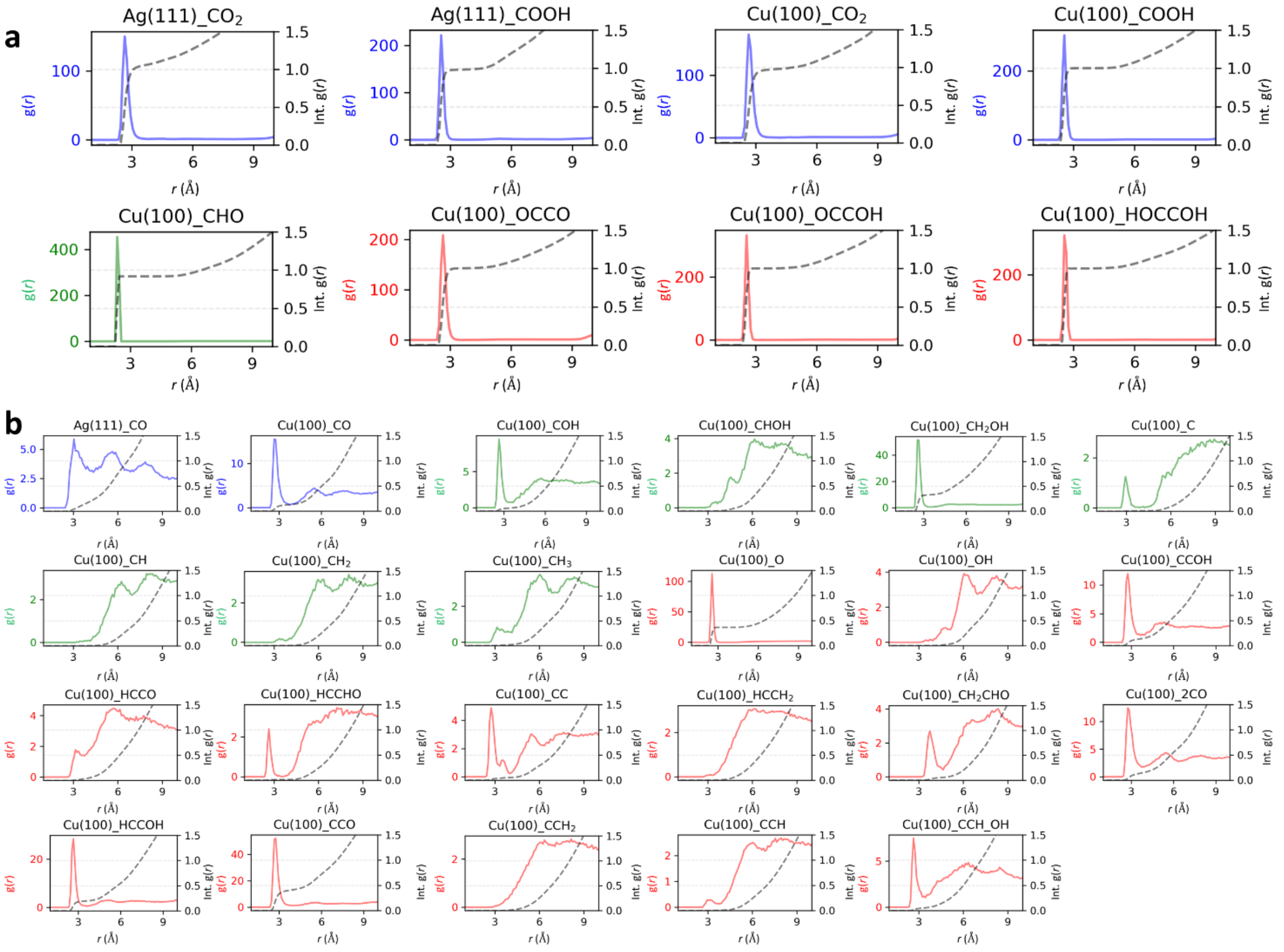

Supplementary Figure 4. Radial distribution function, g(r), calculated using the DFT-CES at −1.0 V_{SHE}. A radial distance, r, is defined between K^+ and O of adsorbates. When the adsorbates have no O, C is used to define r. The integrated (Int.) value of g(r), $\int_0^r 4\pi r'^2 g(r')dr'$, shows the K^+ coordination number (CN) to the adsorbates. The adsorbates are classified into two groups; **a,** ones coordinated by a cation with CN ~ 1, and **b,** the other ones that are not coordinated by a cation, where CN < 1.

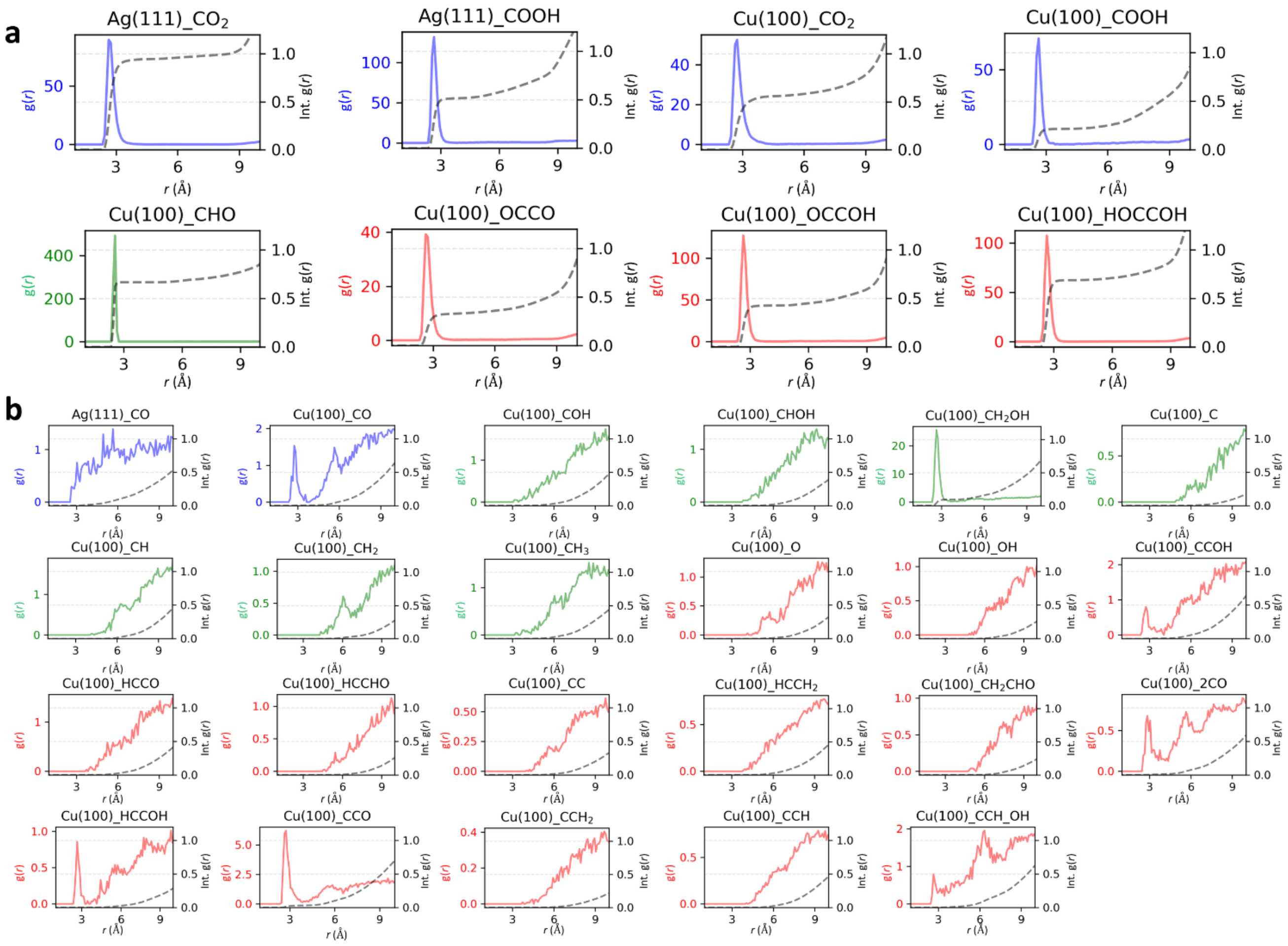

Supplementary Figure 5. Radial distribution function, g(r), calculated using the DFT-CES at −0.5 V_{SHE}, that is the potential at point of zero charge, E_{PZC}. A radial distance, r, is defined between K^+ and O of adsorbates. When the adsorbates have no O, C is used to define r. The integrated (Int.) value of g(r), $\int_0^r 4\pi r'^2 g(r')dr'$, shows the K^+ coordination number (CN) to the adsorbates. **a,b,** Due to the smaller number of K^+ at the E_{PZC}, the calculated CN is generally lower compared to the case of −1.0 V_{SHE}. However, the intermediates coordinated by a cation in the **Supplementary Fig. 4** still show a clear cation-coordinating ability (**a**), while those that are not show CN ~ 0 (**b**).

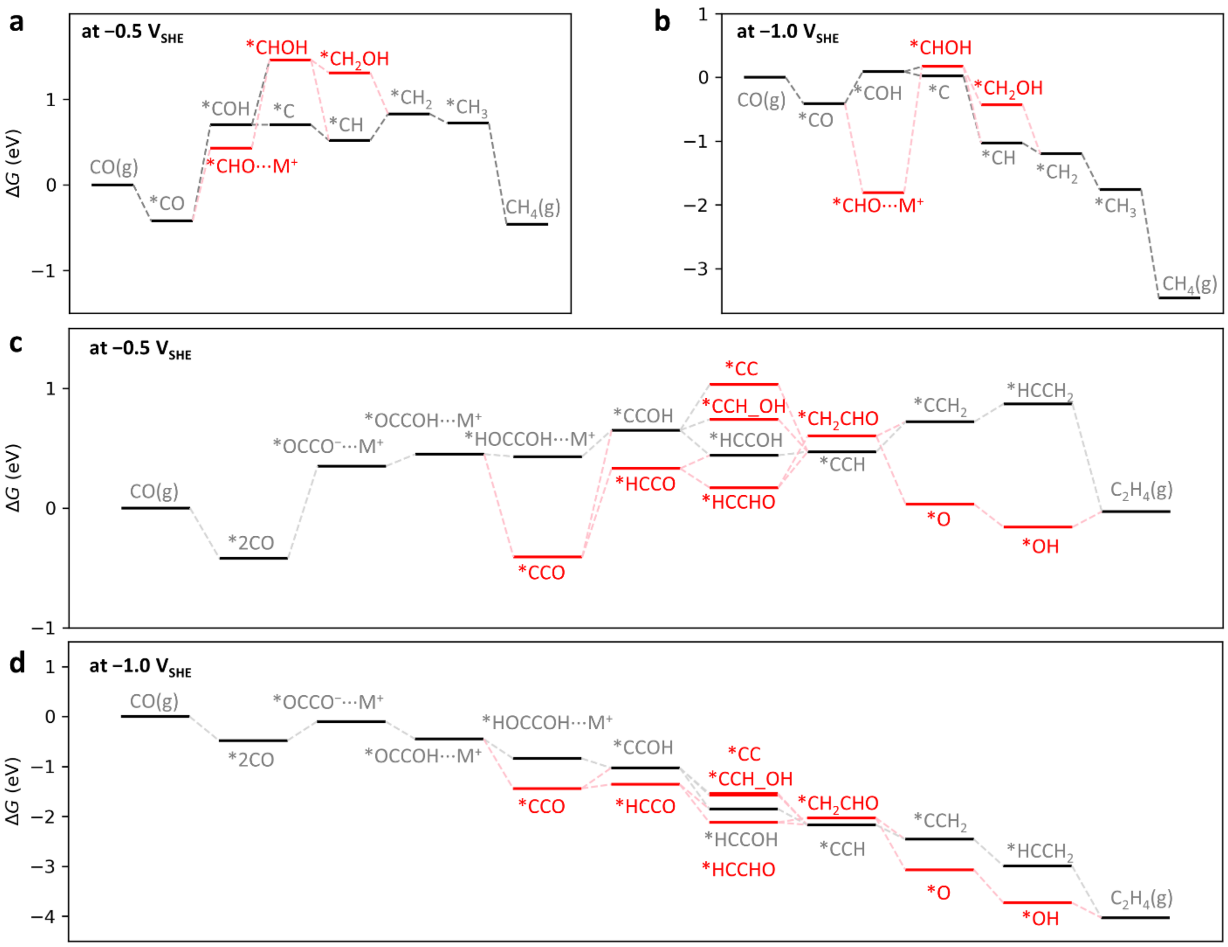

Supplementary Figure 6. Reaction energy diagram for CO-to-CH_4 and CO-to-C_2H_4 reaction paths on Cu(100), calculated using DFT-CES energetics. The reaction free energy (ΔG) is calculated for different reaction paths. The cation-coordinated intermediates are specified by appending $\cdots M^+$. **a,b,** Reaction energy diagrams for the CO-to-CH_4 reaction path at −0.5 V_{SHE} (**a**) and those at −1.0 V_{SHE} (**b**). **c,d,** Reaction energy diagrams for the CO-to-C_2H_4 reaction path at −0.5 V_{SHE} (**c**) and those at −1.0 V_{SHE} (**d**). Energetically feasible reaction paths are highlighted using black solid line, while the other ones are shown in red.

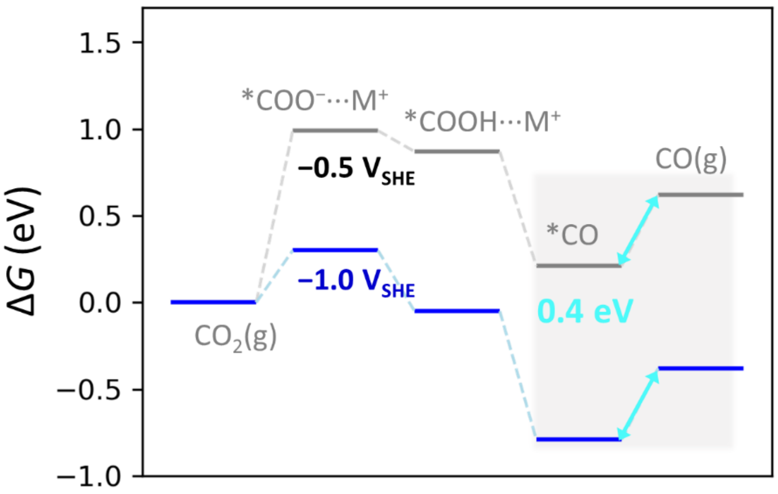

Supplementary Figure 7. Reaction energy diagrams for CO_2-to-CO reaction path on Cu(100), calculated using DFT-CES energetics. The reaction free energy (ΔG) is calculated when −0.5 V_{SHE} is applied (black) and −1.0 V_{SHE} is applied (blue). The cation-coordinated intermediates are specified by appending $\cdots M^+$. The CO desorption step requires *ca.* 0.4 eV of energetic cost.

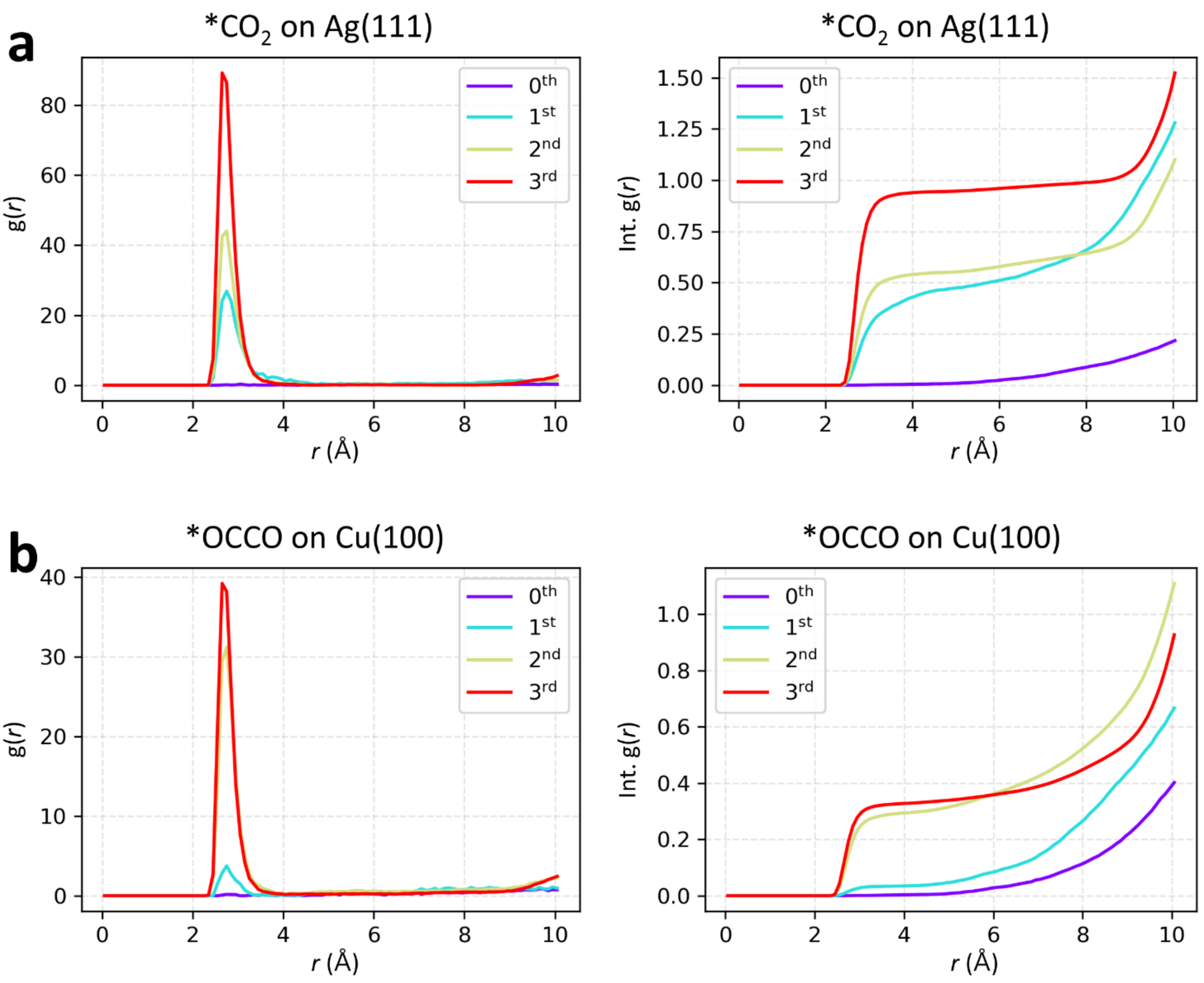

Supplementary Figure 8. Change of cation-coodrinating structure of *CO_2 and *OCCO during DFT-CES iterations. a,b, A radial distance, r, is defined between K^+ and O of *CO_2 on the Ag(111) (**a**) or *OCCO on the Cu(100) (**b**). The radial distribution function, g(r), and the integrated (Int.) value of g(r), $\int_0^r 4\pi r'^2 g(r')dr'$, are shown. Applied potential is −0.5 V_{SHE}.

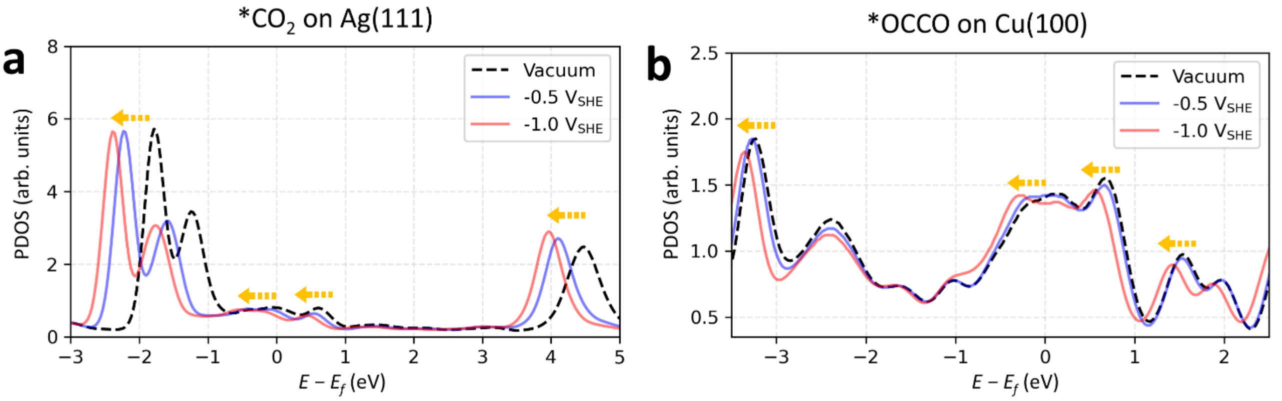

Supplementary Figure 9. Projected density of states (PDOS) calculated using DFT-CES. a, PDOS of CO_2 adsorbate ($^*CO_2$) on Ag(111). **b,** PDOS of OCCO adsorbate (*OCCO) on Cu(100). E_f denotes the Fermi level.

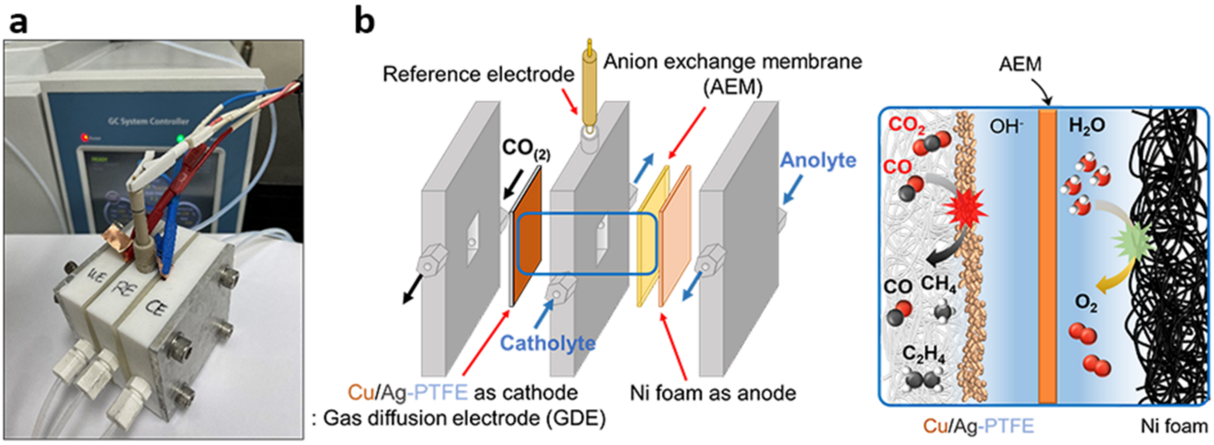

Supplementary Figure 10. Electrochemical flow cell. a, Photograph and **b,** Schematic images of an H-type electrochemical flow cell used for CO_2 and CO electrolysis.

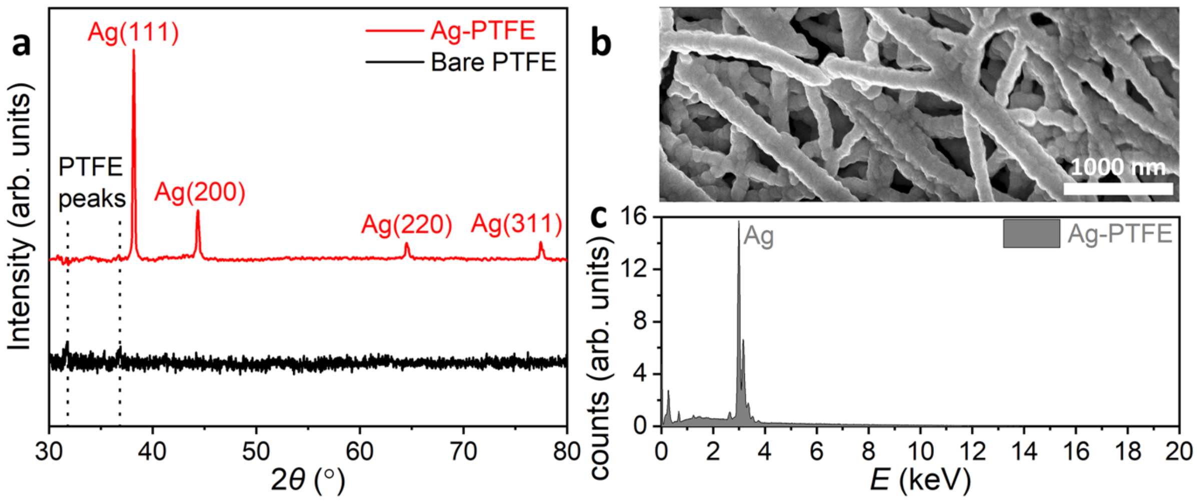

Supplementary Figure 11. Physical characterizations of Ag-PTFE electrode. a, X-ray diffraction (XRD) pattern, **b,** Scanning electron microscope (SEM) image, and **c,** energy-dispersive X-ray spectroscopy (EDS) result of the Ag-PTFE electrode. The XRD result shows clear peaks at 38.1, 44.3, 64.5, and 77.4, which correspond to (111), (200), (220), and (311) facets of polycrystalline Ag. The SEM and EDS results also identify Ag, successfully deposited on the PTFE.

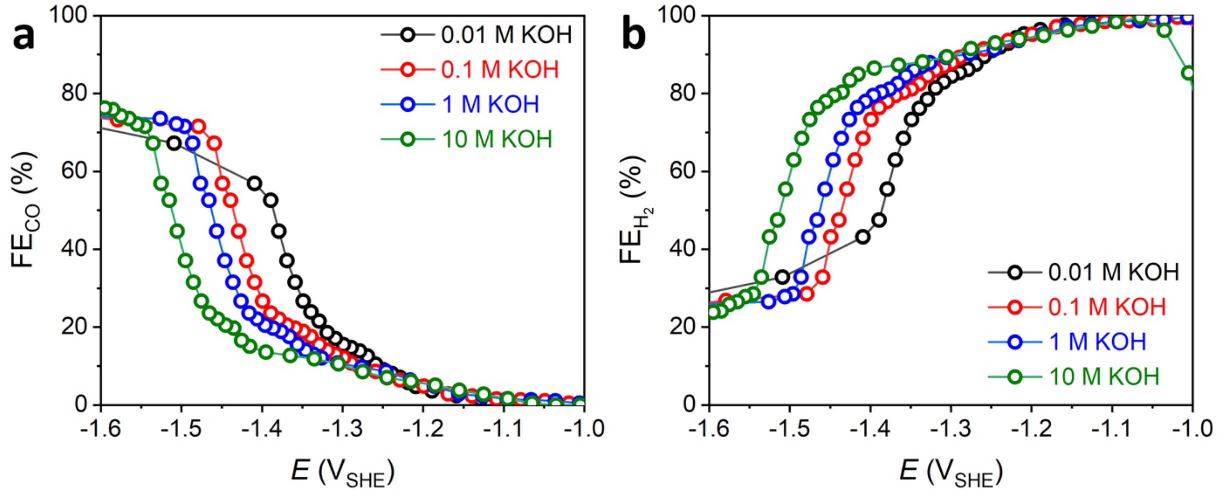

Supplementary Figure 12. Faradaic efficiency (FE) of CO_2RR on the Ag electrode in KOH electrolytes.

a, CO and **b,** H_2 FEs on the Ag electrode measured in 0.01–10 M KOH electrolytes during CO_2RR.

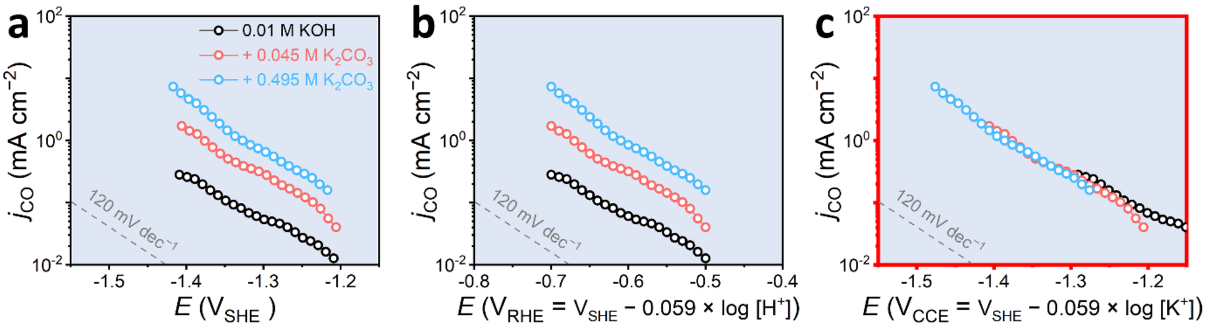

Supplementary Figure 13. CO_2-to-CO conversion on the Ag electrode in KOH + K_2CO_3 electrolytes. The partial current density of CO (j_{CO}) *vs.* potential curves measured on the Ag electrode in an electrochemical flow cell. The electrolytes were 0.01 M KOH with or without additional K_2CO_3 salt (0.045 and 0.495 M). The polarization curves are plotted with respect to the **a,** SHE, **b,** RHE ($V_{SHE} - 0.059 \times \log[H^+]$), or **c,** CCE ($V_{SHE} - 0.059 \times \log[K^+]$) scales. The grey lines indicate a Tafel slope (typically plotted as an inverse function of the present polarization curve) of 120 mV dec^{-1}. A collapse of the polarization curves is found upon the CCE scale, highlighted with a red box.

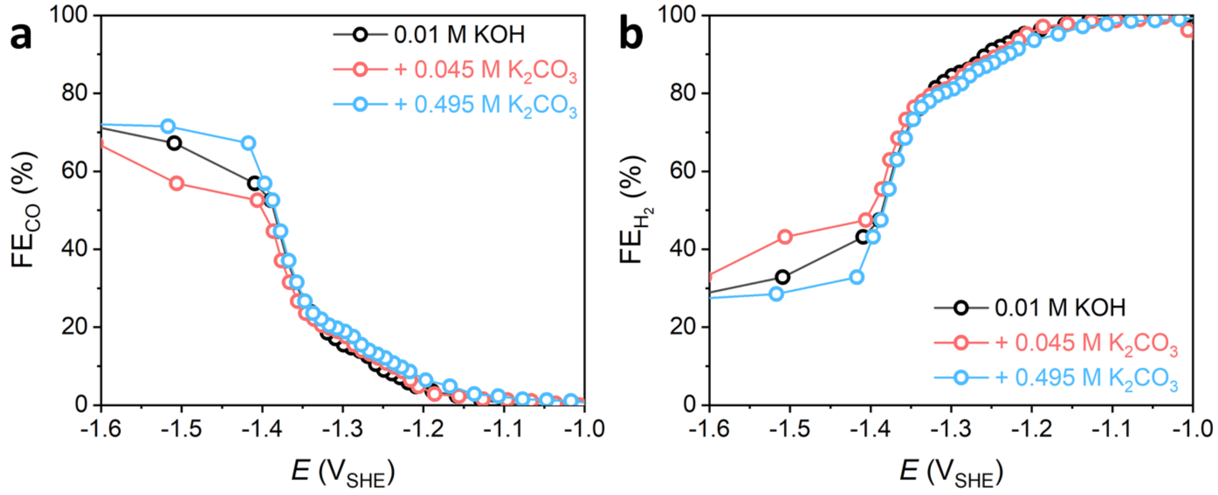

Supplementary Figure 14. Faradaic efficiency (FE) of CO_2RR on the Ag electrode in KOH + K_2CO_3 electrolytes. a, CO and **b,** H_2 FEs on the Ag electrode measured in 0.01 M KOH + 0, 0.045, and 0.495 M K_2CO_3 electrolytes during CO_2RR.

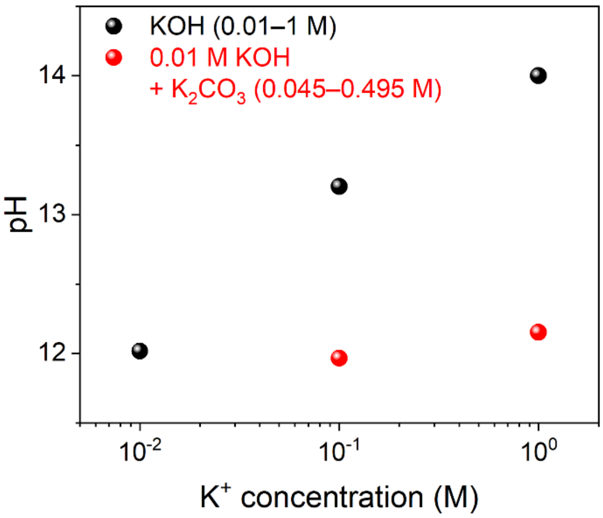

Supplementary Figure 15. Electrolyte pHs. The pH of electrolytes used for CO_2RR on the Ag electrode. KOH electrolytes show an increase in pH with increasing K^+ concentration, while 0.01 M KOH electrolytes with and without additional K_2CO_3 salt (0.045 and 0.495 M) reveal an almost untouched pH value.

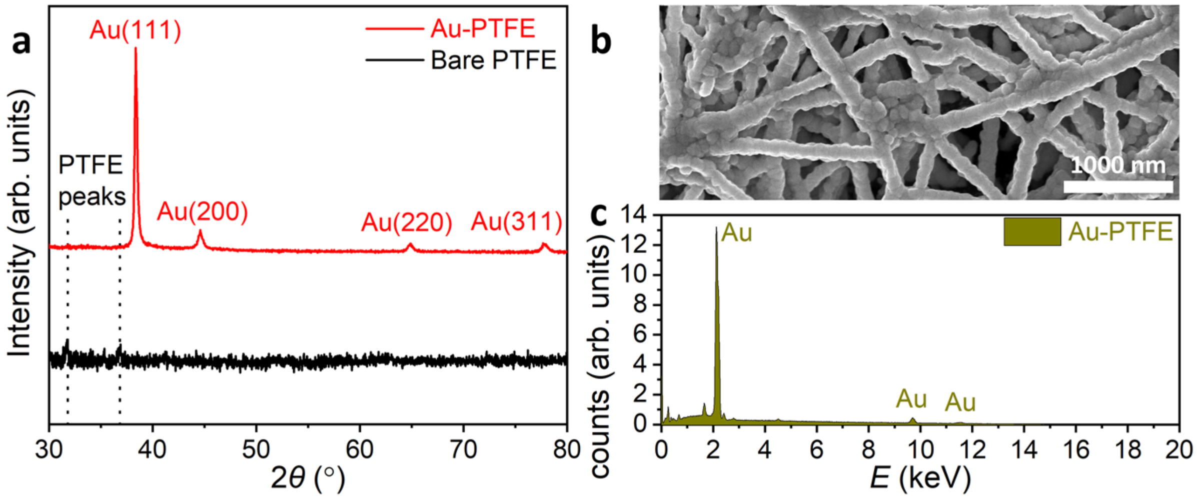

Supplementary Figure 16. Physical characterizations of Au-PTFE electrode. a, XRD pattern, **b,** SEM image, and **c,** EDS result of the Au-PTFE electrode. The XRD result shows clear peaks at 38.3, 44.6, 64.8, and 77.7, which correspond to (111), (200), (220), and (311) facets of polycrystalline Au. The SEM and EDS results also identify Au, successfully deposited on the PTFE.

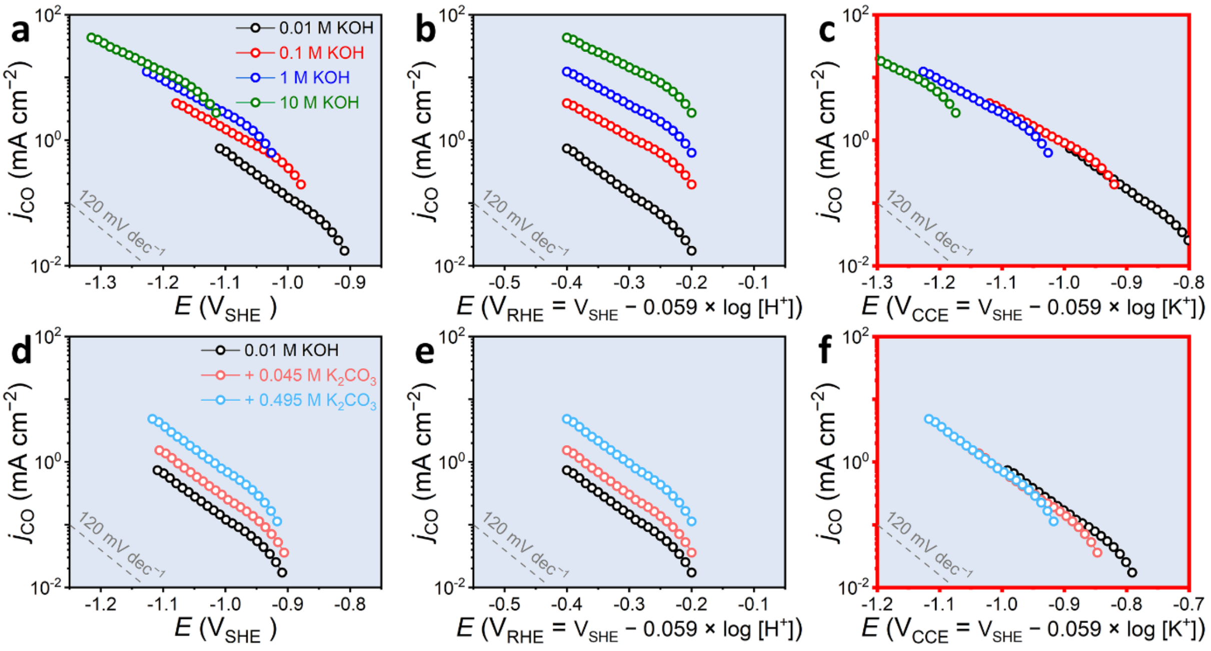

Supplementary Figure 17. CO_2-to-CO conversion on the Au electrode. The j_{CO} *vs*. potential curves measured on the Au electrode in an electrochemical flow cell. The electrolytes were **a–c,** 0.01–10 M KOH and **d–f,** 0.01 M KOH + 0, 0.045, and 0.495 M K_2CO_3. The polarization curves are plotted with respect to the **a,d,** SHE, **b,e,** RHE (V_{SHE} − 0.059 × log[H^+]), or **c,f,** CCE (V_{SHE} − 0.059 × log[K^+]) scales. The grey lines indicate a Tafel slope (typically plotted as an inverse function of the present polarization curve) of 120 mV dec^{-1}. A collapse of the polarization curves is found upon the CCE scale, highlighted with a red box.

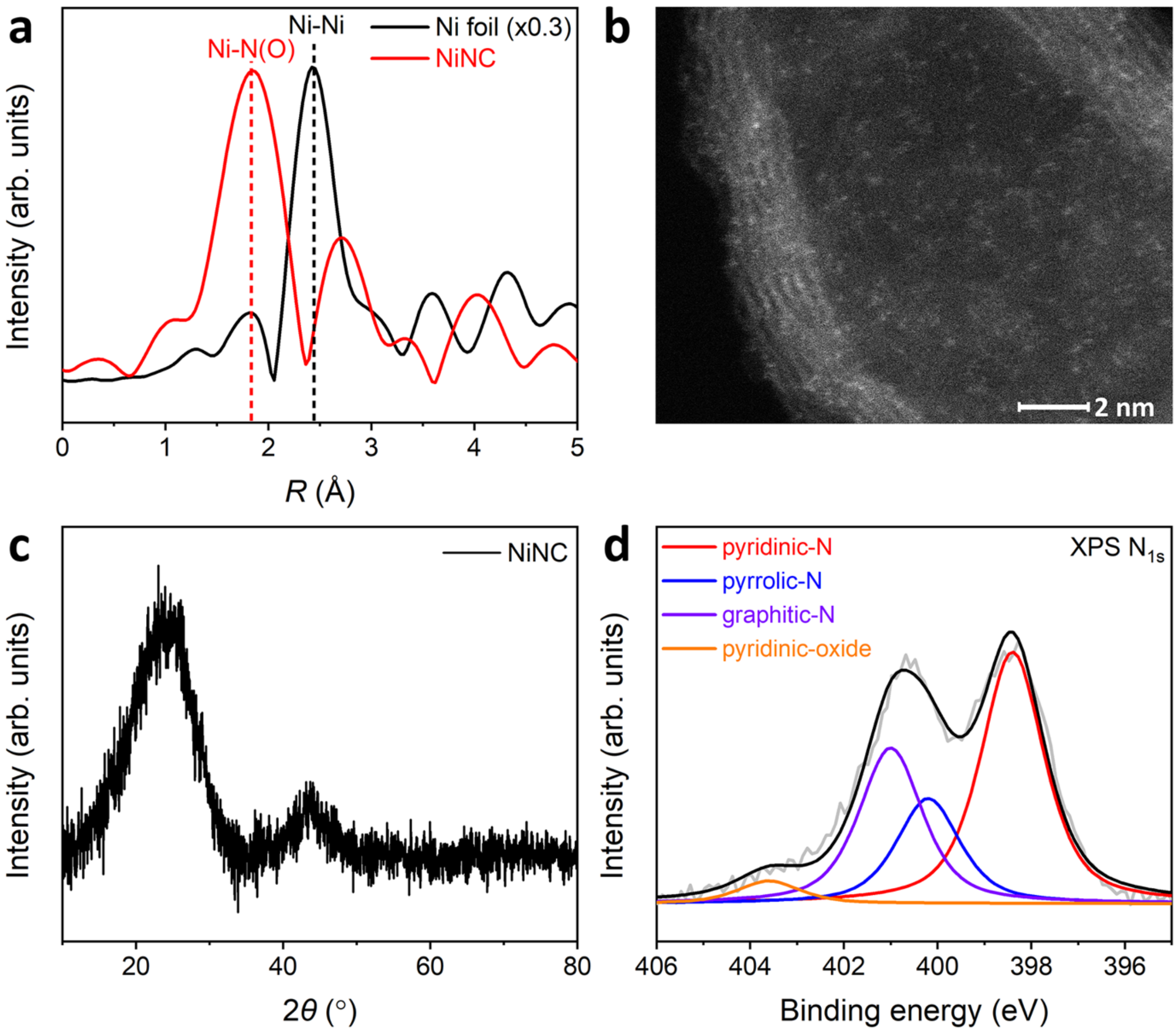

Supplementary Figure 18. Physical characterizations of the NiNC electrode. a, Extended X-ray absorption fine structure (EXAFS) spectrum, **b,** high-angle annular dark-field scanning transmission electron microscopy (HAADF-STEM) image, **c,** XRD pattern, and **d,** X-ray photoelectron spectroscopy (XPS) N_{1s} spectrum of the NiNC electrode. The EXAFS spectrum shows the presence of a strong Ni-N(O) interaction, without Ni-Ni interaction, and the HAADF-STEM image reveals atomically dispersed Ni species without discernible Ni clusters. In addition, the XRD result confirms the successful carbonization of the precursor mixture during pyrolysis, and XPS identifies the functionalization of the carbon support with N moieties, which can act as anchoring sites for the stabilization of the isolated Ni ions. Therefore, the results confirm the successful preparation of the NiNC catalyst with abundant isolated Ni species, highly active towards CO_2RR.

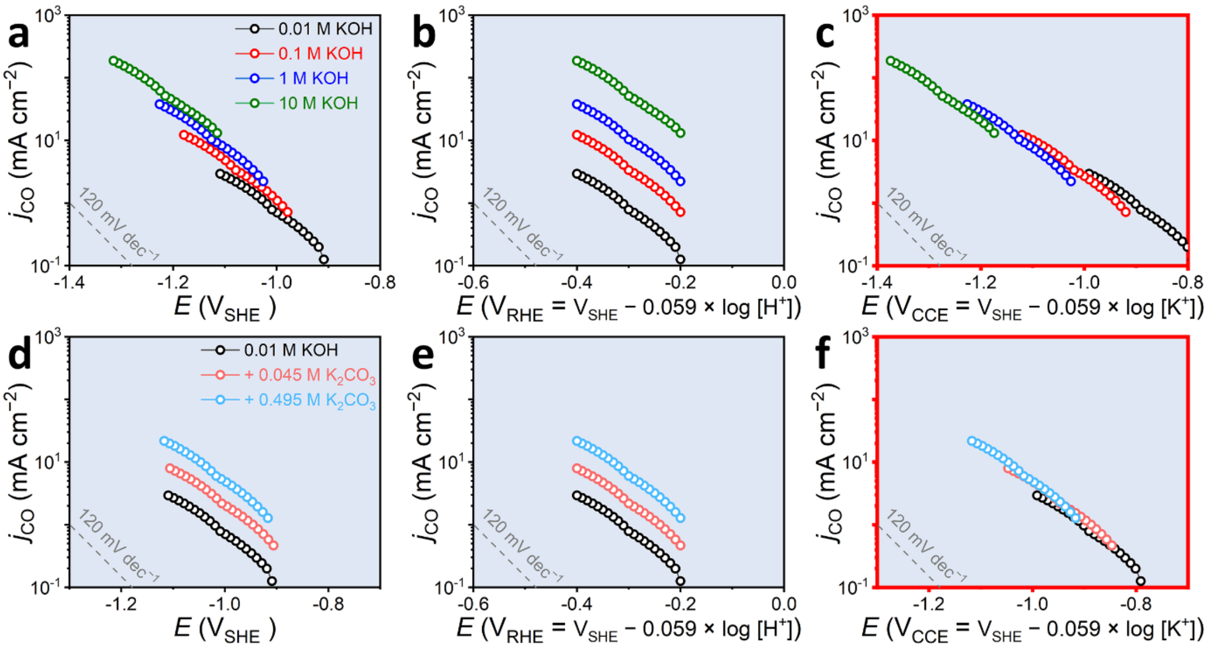

Supplementary Figure 19. CO_2-to-CO conversion on the NiNC electrode. The j_{CO} *vs.* potential curves measured on the NiNC electrode in an electrochemical flow cell. The electrolytes were **a–c,** 0.01–10 M KOH and **d–f,** 0.01 M KOH + 0, 0.045, and 0.495 M K_2CO_3. The polarization curves are plotted with respect to the **a,d,** SHE, **b,e,** RHE (V_{SHE} − 0.059 × log[H^+]), or **c,f** CCE (V_{SHE} − 0.059 × log[K^+]) scales. The grey lines indicate a Tafel slope (typically plotted as an inverse function of the present polarization curve) of 120 mV dec^{-1}. A collapse of the polarization curves is found upon the CCE scale, highlighted with a red box.

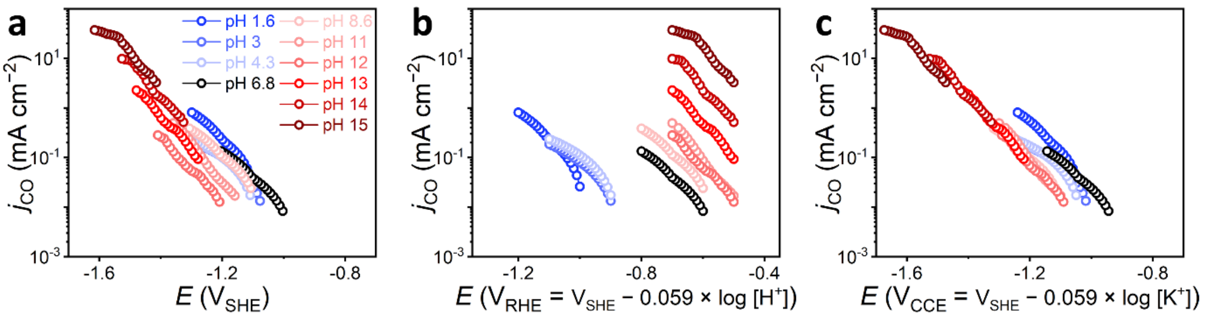

Supplementary Figure 20. CO_2-to-CO conversion on the Ag electrode in acid, neutral, and alkaline electrolytes. The j_{CO} *vs.* potential curves measured on the Ag electrode in an electrochemical flow cell. The CO_2RR polarization curves are plotted with respect to **a,** SHE, **b,** RHE (V_{SHE} − 0.059 × log[H^+]), or **c,** CCE (V_{SHE} − 0.059 × log[K^+]) scales, respectively. Electrolytes were 0.03 M $HClO_4$ + 0.07 M $KClO_4$ for pH 1.6, 0.03 M H_3PO_4 + 0.07 M KH_2PO_4 M for pH 3, 0.1 M KH_2PO_4 for pH 4.3, CO_2-saturated 0.1 M $KHCO_3$ for pH 6.8, Ar-saturated 1.5 M $KHCO_3$ for pH 8.6, 0.05 M K_2CO_3 for pH 11, 0.01–10 M KOH for pH 12–15. The results reveal that clear collapse of the polarization curves upon the CCE scale is limitedly found at pH higher than 8.6, indicating that the CCET may hardly govern the RDS of CO_2RR in neutral and acidic environments.

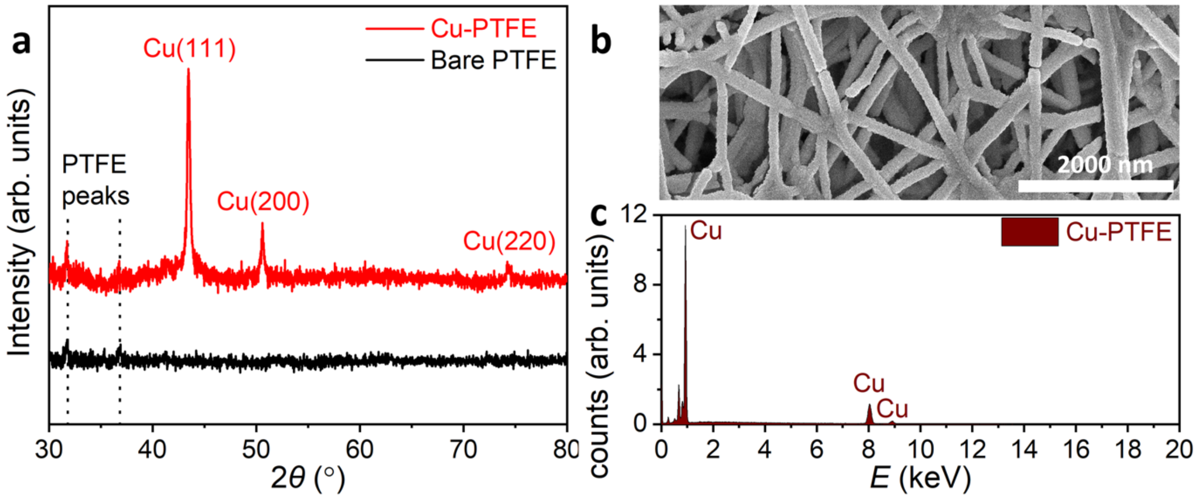

Supplementary Figure 21. Physical characterizations of Cu-PTFE electrode. a, XRD pattern, **b,** SEM image, and **c,** EDS result of the Cu-PTFE electrode. The XRD result shows clear peaks at 43.2, 50.4, and 74.1, which correspond to (111), (200), and (220) facets of polycrystalline Cu. The SEM and EDS results also identify Cu, successfully deposited on the PTFE.

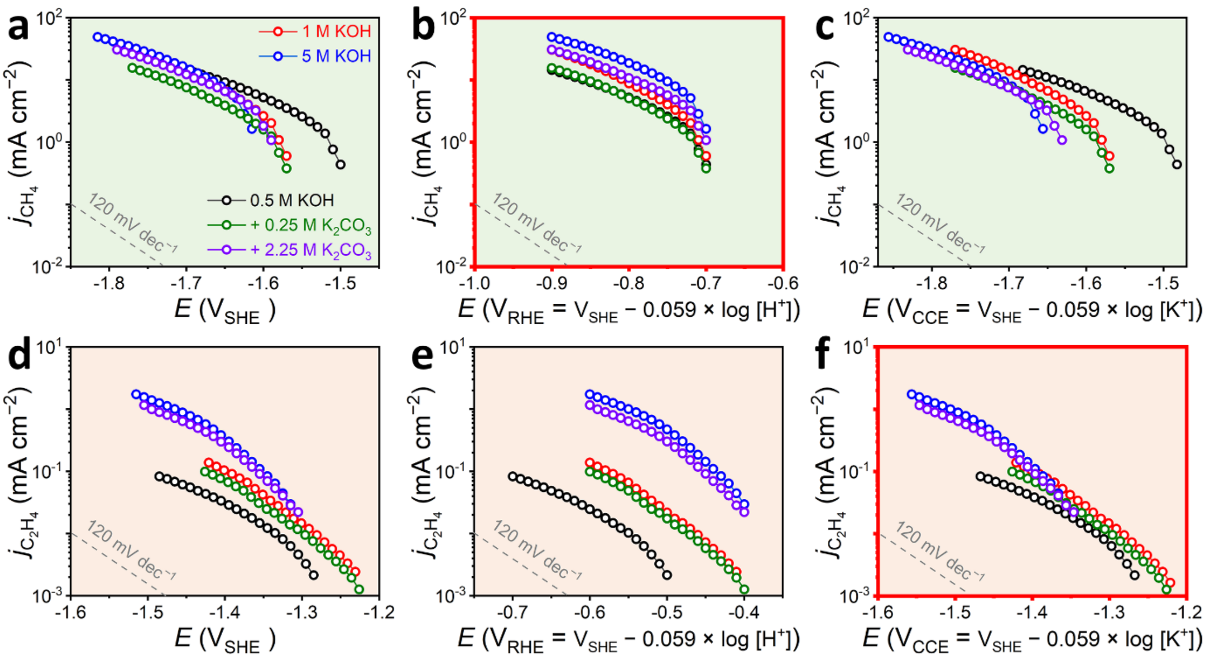

Supplementary Figure 22. Electrochemical CO_2RR on the Cu electrode. The **a–c,** partial current density of methane (j_{CH4}) and **d–f,** partial current density of ethylene (j_{C2H4}) *vs.* potential curves measured on the Cu electrode in an electrochemical flow cell. The electrolytes were 0.5–5 M KOH and 0.5 M KOH + 0.25 and 2.25 M K_2CO_3. The polarization curves are plotted with respect to the **a,d,** SHE, **b,e,** RHE (V_{SHE} − 0.059 × log[H^+]), or **c,f,** CCE (V_{SHE} − 0.059 × log[K^+]) scales. The grey lines indicate a Tafel slope (typically plotted as an inverse function of the present polarization curve) of 120 mV dec^{-1}. The red boxes indicate the potential scales, at which a collapse of polarization curves was found in CORR for CH_4 and C_2H_4 formations (**Fig. 3d–i**). Unlike the CORR results, the CO_2RR polarization curves show non-negligible departures upon all potential scales for both CH_4 and C_2H_4 formations. Despite a relatively better collapse of j_{C2H4} of CO_2RR upon the CCE scale, a correlation between j_{C2H4} and $|\sigma|$ at −1.4 V_{SHE} reveals a poor R^2 value of 0.68 (**Supplementary Fig. 25**), much lower than 0.98 for CORR **(Fig. 4c)**. Therefore, as discussed in **Supplementary Note 2**, CORR was chosen as a main model reaction in this study for clearer elucidation of the role of the alkali metal cations in C–C coupling.

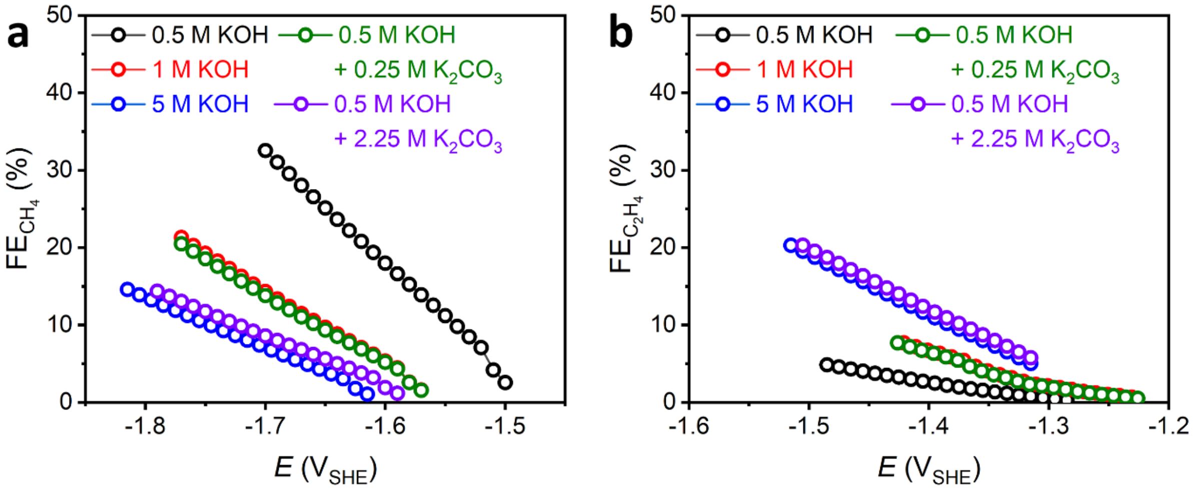

Supplementary Figure 23. Faradaic efficiency (FE) of CO_2RR on the Cu electrode. a, CH_4 and **b,** C_2H_4 FEs on the Cu electrode during CO_2RR. The electrolytes were 0.5–5 M KOH and 0.5 M KOH + K_2CO_3 salt (0.25 and 2.25 M).

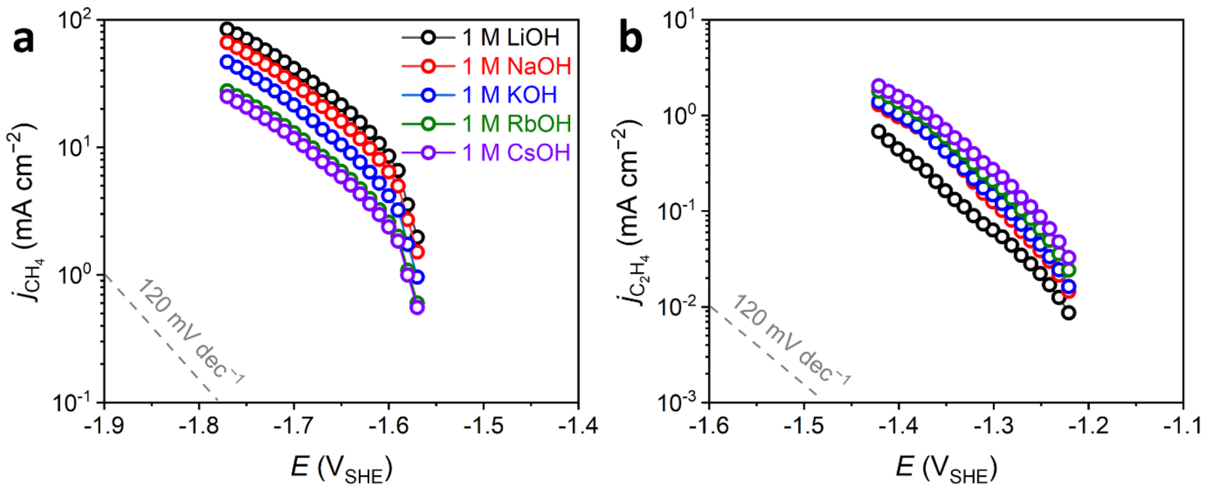

Supplementary Figure 24. Electrochemical CO_2RR on the Cu electrode in various MOH electrolytes. The **a,** j_{CH4} and **b,** j_{C2H4} *vs.* potential curves measured on the Cu electrode in an electrochemical flow cell. The electrolyte was 1 M MOH with various M^+ identity of Li^+, Na^+, K^+, Rb^+, and Cs^+. The polarization curves are plotted with respect to the SHE scale. The grey lines indicate a Tafel slope (typically plotted as an inverse function of the present polarization curve) of 120 mV dec^{-1}. The results show that both CH_4 and C_2H_4 formations are affected by cation identities: decreasing CH_4 formation but increasing C_2H_4 formation as M^+ size becomes larger. This finding is qualitatively consistent with the results from CORR (**Supplementary Fig. 29**).

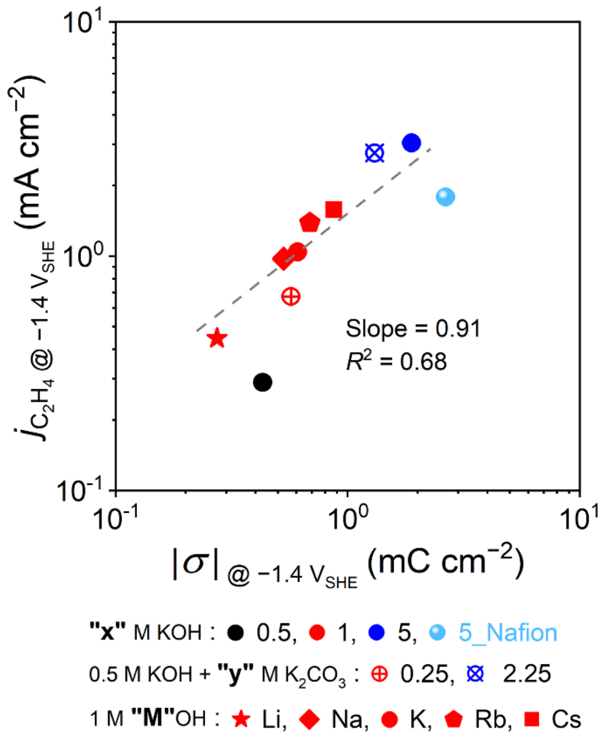

Supplementary Figure 25. Reaction kinetic study of CO_2RR on the Cu electrode. A correlation plot between j_{C2H4} and $|\sigma|$. Their partial current densities and $|\sigma|$ values were collected at −1.4 V_{SHE}. Fitting values and guideline (dashed line) are also shown in the figure. The electrolytes used for gathering these data can be classified into three different categories: KOH electrolytes with different concentrations (filled circles), 0.5 M KOH electrolytes with additional K_2CO_3 salt (crossed circles), and 1 M MOH electrolytes (filled symbols), where M is Li, Na, K, Rb, and Cs. In addition, the data collected on the Nafion-coated Cu electrode in 5 M KOH electrolyte (a filled circle with pale blue color) is also provided. Here, the total M^+ concentration in the electrolytes and M^+ identity are distinguished by color and symbol shape, respectively.

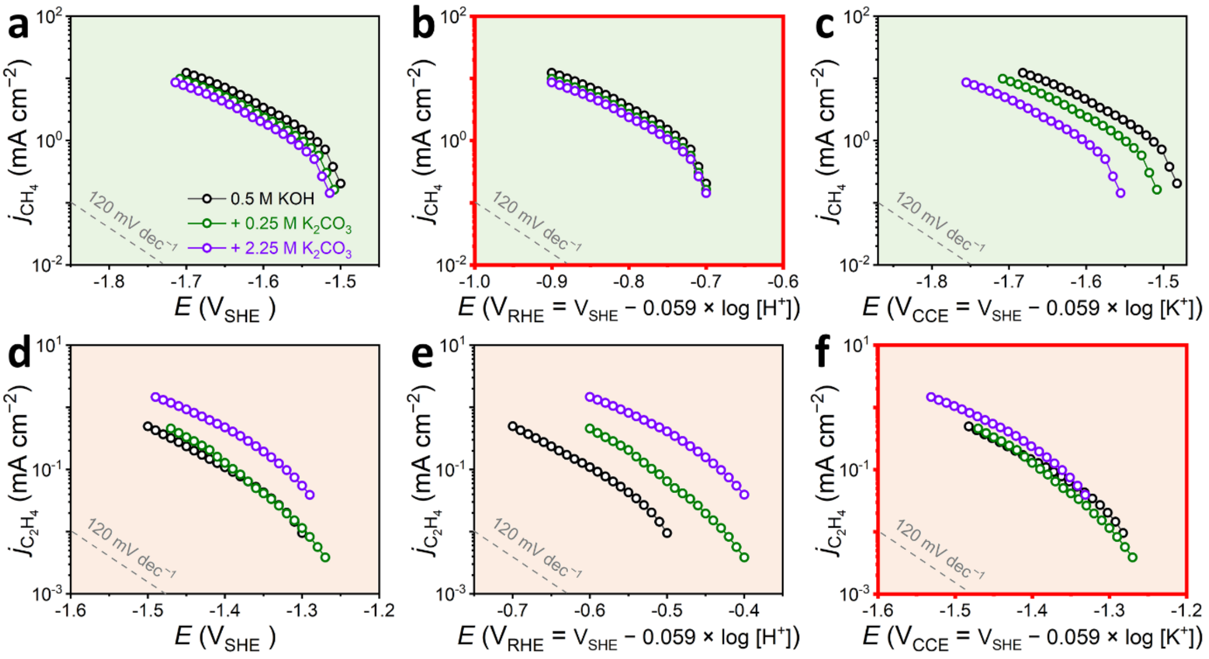

Supplementary Figure 26. Electrochemical CORR on the Cu electrode in KOH + K_2CO_3 electrolytes. The **a–c,** j_{CH4} and **d–f,** j_{C2H4} *vs.* potential curves measured on the Cu electrode in an electrochemical flow cell. The electrolytes were 0.5 M KOH with or without additional K_2CO_3 salt (0.25 and 2.25 M). The polarization curves are plotted with respect to the **a,d,** SHE, **b,e,** RHE ($V_{SHE} - 0.059 \times \log[H^+]$), or **c,f,** CCE ($V_{SHE} - 0.059 \times \log[K^+]$) scales. The grey lines indicate a Tafel slope (typically plotted as an inverse function of the present polarization curve) of 120 mV dec^{-1}. A collapse of polarization curves was found for CH_4 and C_2H_4 formations upon the RHE and CCE scales, respectively, highlighted with a red box.

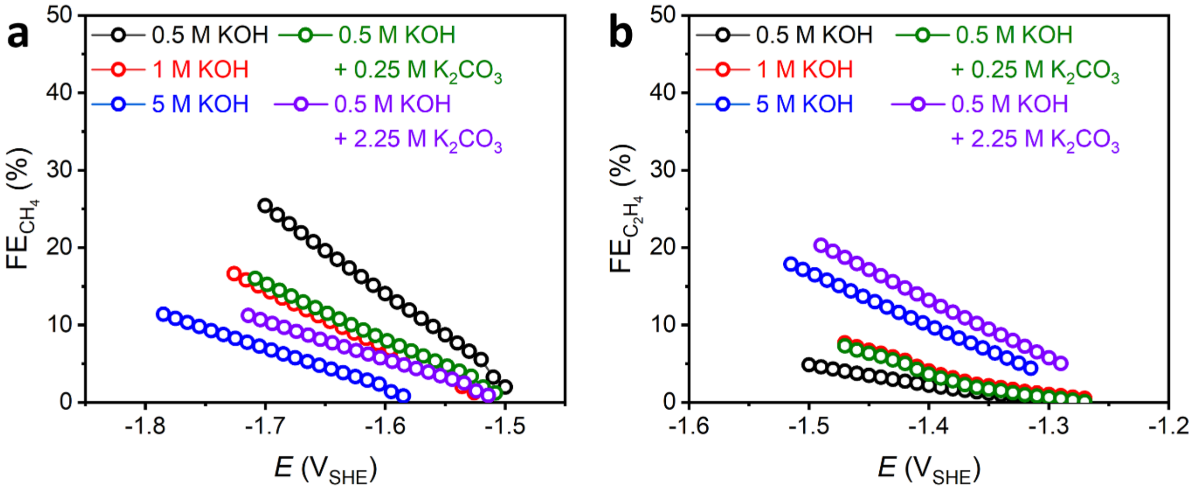

Supplementary Figure 27. Faradaic efficiency (FE) of CORR on the Cu electrode. a, CH_4 and **b,** C_2H_4 FEs on the Cu electrode during CORR. The electrolytes were 0.5–5 M KOH and 0.5 M KOH + K_2CO_3 salt (0.25 and 2.25 M).

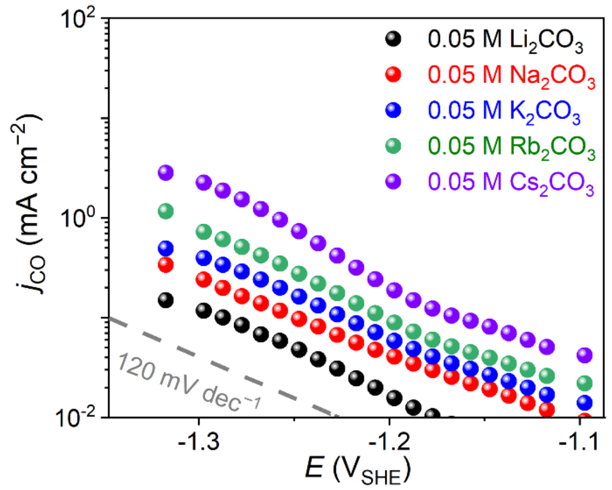

Supplementary Figure 28. Electrochemical CO_2RR on the Ag electrode in various M_2CO_3 electrolytes. The j_{CO} *vs.* potential curves measured on the Ag electrode in an electrochemical flow cell. The electrolyte was 0.05 M M_2CO_3 with various M^+ identity of Li^+, Na^+, K^+, Rb^+, and Cs^+. The polarization curves are plotted with respect to the SHE scale. A grey line indicates a Tafel slope (typically plotted as an inverse function of the present polarization curve) of 120 mV dec^{-1}. The result shows that CO formation is affected by cation identities: increasing CO formation as M^+ size becomes larger.

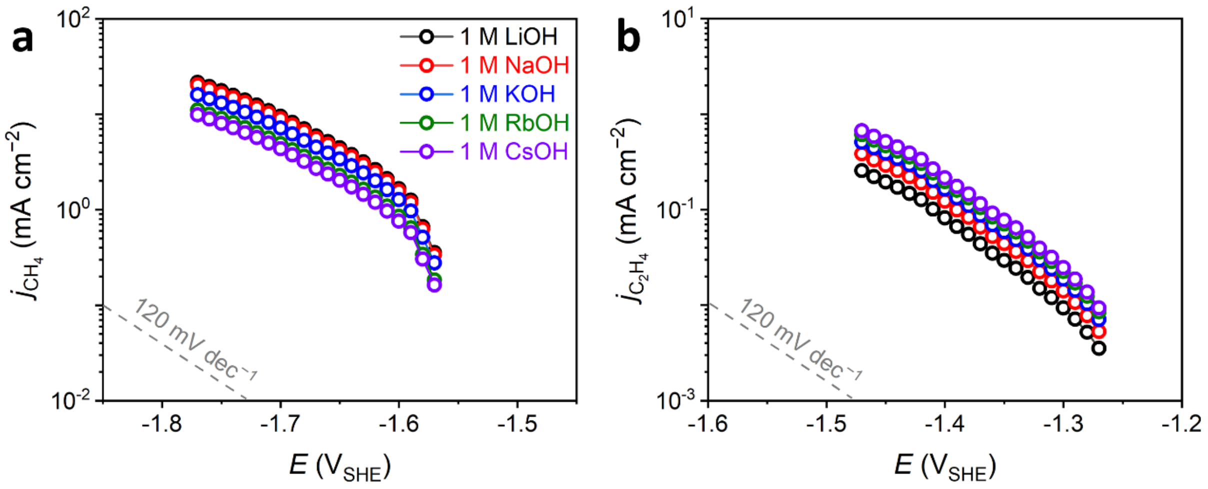

Supplementary Figure 29. Electrochemical CORR on the Cu electrode in various MOH electrolytes. The **a,** j_{CH4} and **b,** j_{C2H4} *vs.* potential curves measured on the Cu electrode in an electrochemical flow cell. The electrolyte was 1 M MOH with various M^+ identity of Li^+, Na^+, K^+, Rb^+, and Cs^+. The polarization curves are plotted with respect to the SHE scale. The grey lines indicate a Tafel slope (typically plotted as an inverse function of the present polarization curve) of 120 mV dec^{-1}. The results show that both CH_4 and C_2H_4 formations are affected by cation identities: decreasing CH_4 formation but increasing C_2H_4 formation as M^+ size becomes larger.

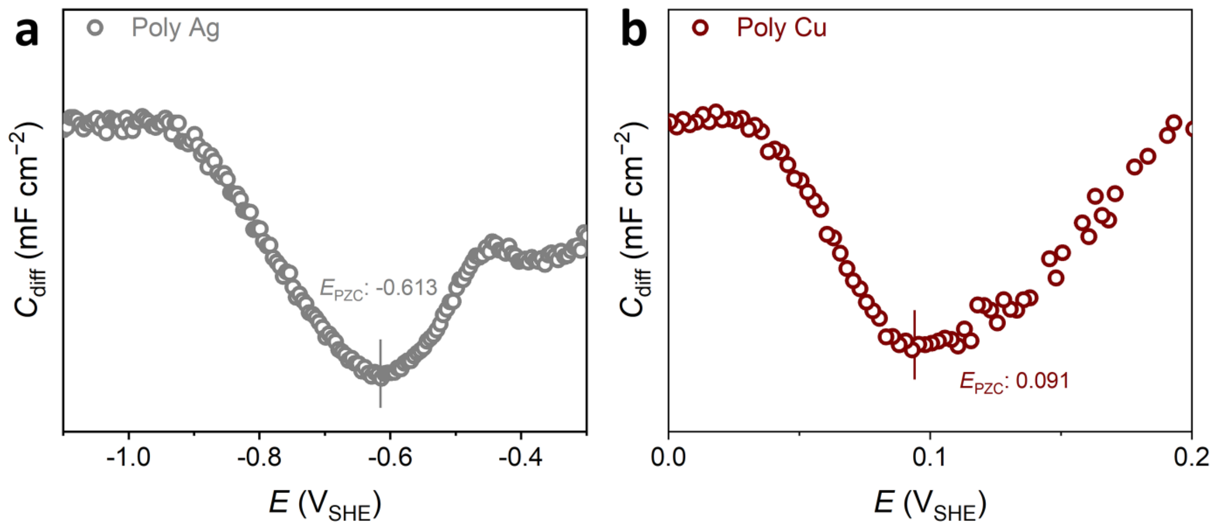

Supplementary Figure 30. E_{PZC} measurements. E_{PZC} of the **a,** Ag and **b,** Cu electrodes. C_{diff} curves were measured in 2 mM NaF electrolyte by a staircase potentio electrochemical impedance spectroscopy (SPEIS). E_{PZC} of the Ag and Cu electrodes are determined as *ca.* −0.613 and 0.091 V_{SHE}, respectively, at which the C_{diff} has a minimum value. The results correspond well to the E_{PZC} values reported in the literature (Ag: −0.584 V_{SHE}, Cu: 0.09 V_{SHE})[31].

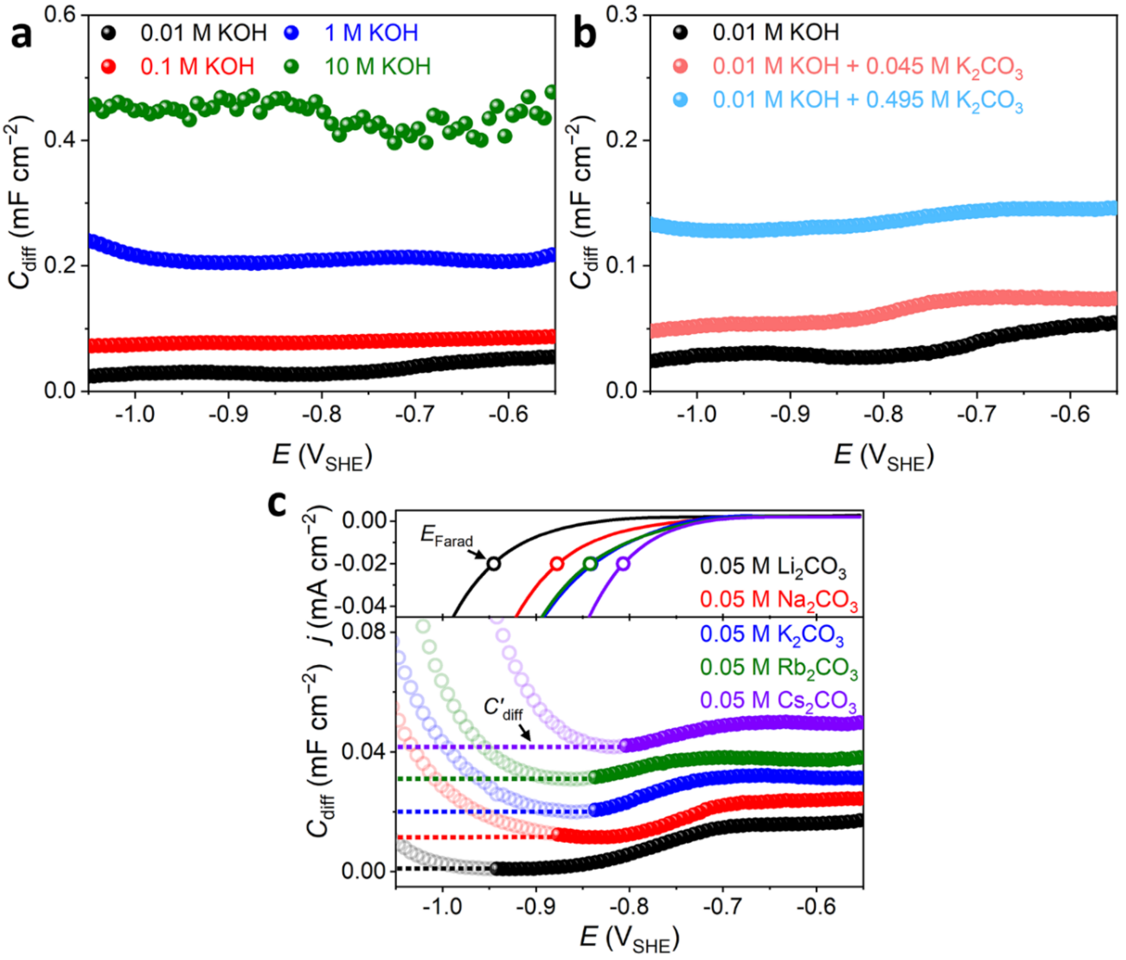

Supplementary Figure 31. C_{diff} curves of the Ag electrode. The C_{diff} curves of the Ag electrode measured in **a,** 0.01–10 M KOH, **b,** 0.01 M KOH + 0, 0.045, and 0.495 M K_2CO_3, and **c,** 0.05 M M_2CO_3 (M = Li, Na, K, Rb, and Cs) electrolytes. For two former cases, the $|\sigma|$ at a certain potential (E' *vs.* SHE) was estimated by integrating the C_{diff} curve from the E_{PZC} of Ag, −0.613 V_{SHE}, to the E'. However, for the 0.05 M M_2CO_3 electrolytes, non-negligible Faradaic reactions (*e.g.*, hydrogen evolution reaction) occur on the Ag electrode (upper panel in **c**)[32]. Unfortunately, the Faradaic charge leads to an artifact in the C_{diff} measurements and a considerable increase in the C_{diff} value at the high cathodic potential range (lower panel in **c**). To estimate the $|\sigma|$, we thus assumed that the C_{diff} curve follows the Gouy-Chapman-Stern model, which predicts a constant C_{diff} value at a potential far from E_{PZC}. Namely, we set the C_{diff} as a constant value (C'_{diff}, dotted lines) at a potential below an onset potential of the Faradaic reaction (E_{Farad}), defined here as the potential corresponding to $j = -0.02$ mA cm^{-2}. The $|\sigma|$ at a certain potential (E' *vs.* SHE) was thus estimated by the following equation,

$$|\sigma| = |\int_{E_{PZC}}^{E_{Farad}} C_{diff}\, dE\,| + |\int_{E_{Farad}}^{E'} C'_{diff}\, dE\,|.$$

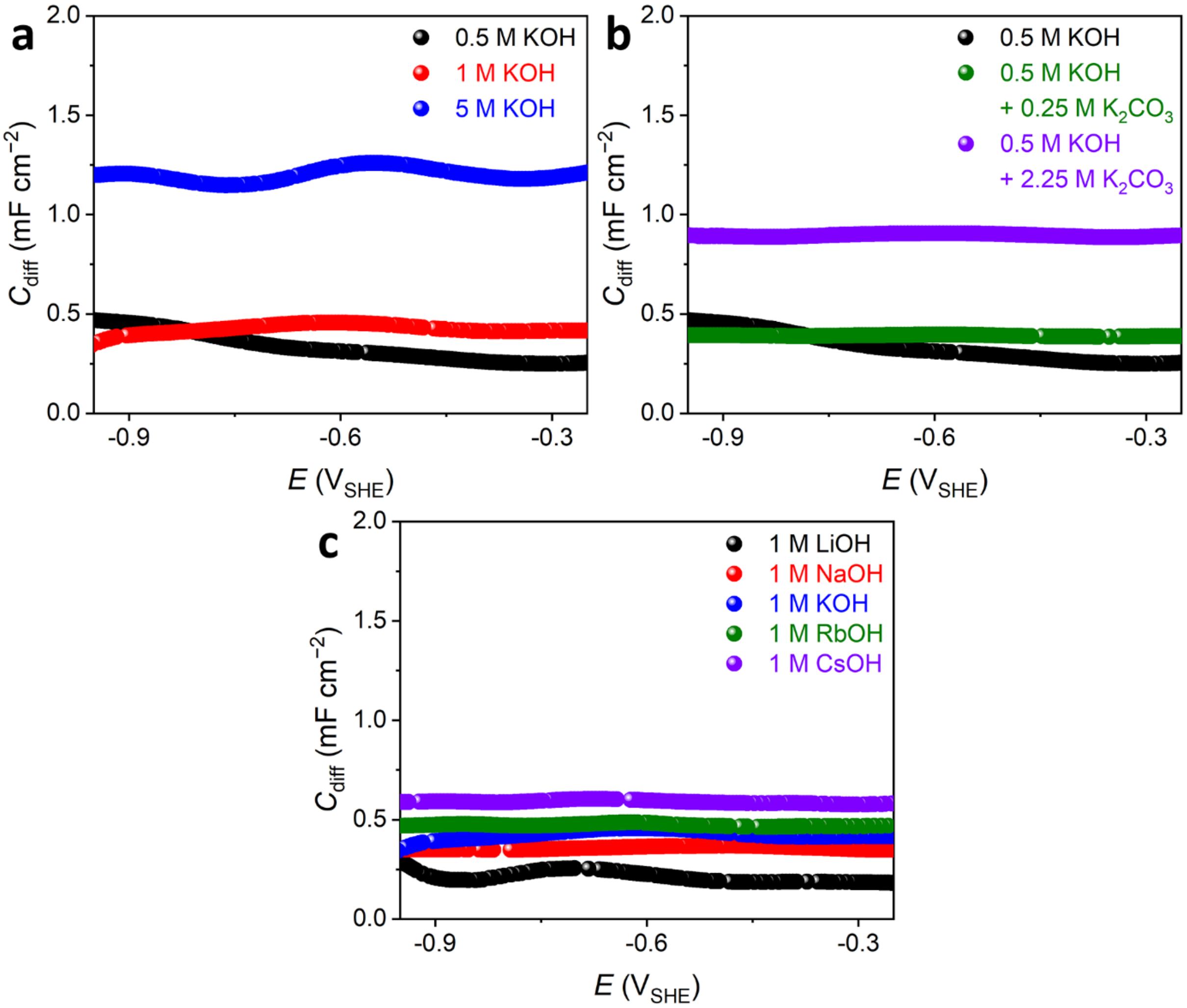

Supplementary Figure 32. C_{diff} curves of the Cu electrode. The C_{diff} curves of the Cu electrode measured in **a,** 0.5–5 M KOH, **b,** 0.5 M KOH + 0, 0.25, and 2.25 M K_2CO_3, and **c,** 1 M MOH (M = Li, Na, K, Rb, and Cs) electrolytes. The $|\sigma|$ at a certain potential (E' *vs.* SHE) was estimated by integrating the C_{diff} curve from the E_{PZC} of Cu, 0.091 V_{SHE}, to the E'.

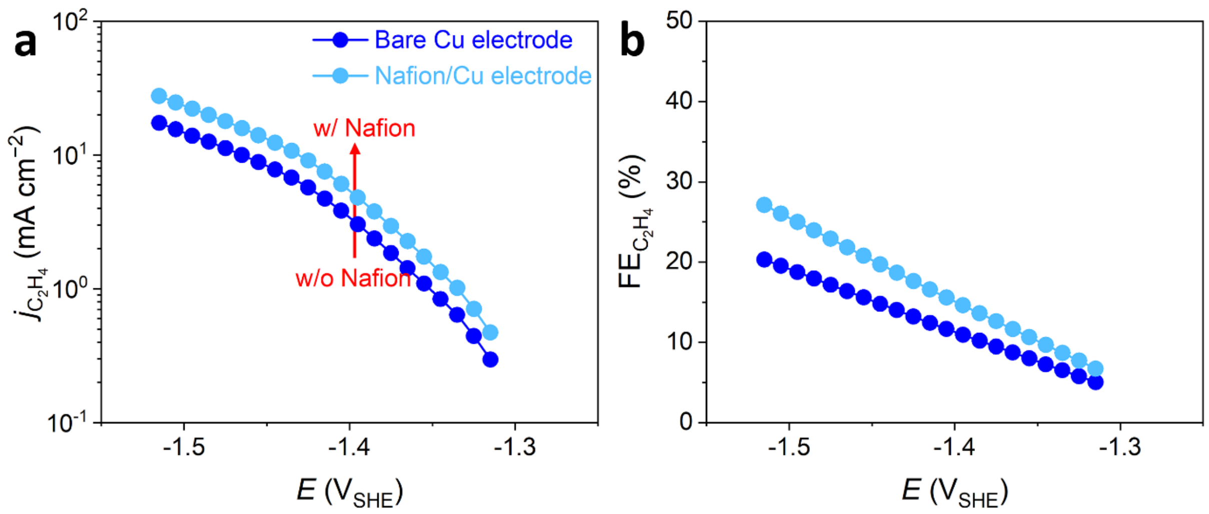

Supplementary Figure 33. Ionomer effects on the CO_2RR. The **a,** j_{C2H4} and **b,** C_2H_4 FE *vs.* potential curves measured on the bare and Nafion-coated Cu electrodes during CO_2RR. The electrolyte was 5 M KOH.

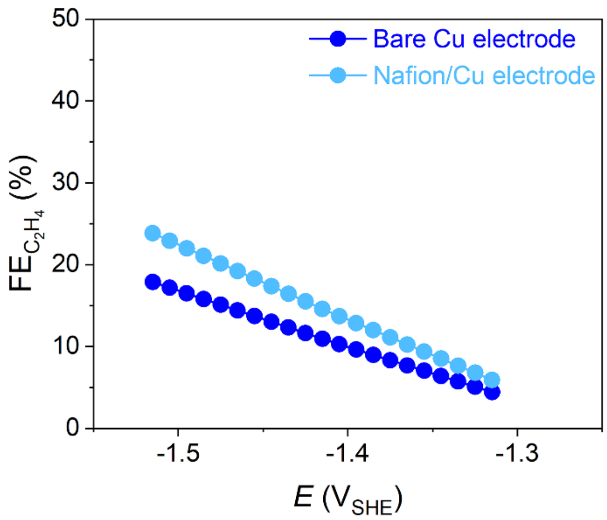

Supplementary Figure 34. Ionomer effects on the CORR. The C_2H_4 FE *vs.* potential curve measured on the bare and Nafion-coated Cu electrodes during CORR. The electrolyte was 5 M KOH.

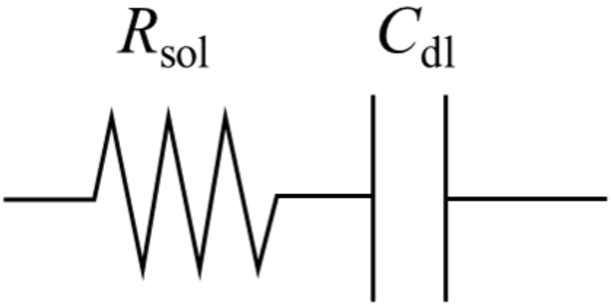

Supplementary Figure 35. RC circuit for fitting obtained impedance data. The obtained impedance data was fitted by the RC circuit given as Z = $R + 1/i\omega C_{diff}$, where R is the solution resistance, and ω is the circular frequency.

Supplementary References

1. Lim, H.-K., Lee, H. & Kim, H. A seamless grid-based interface for mean-field QM/MM coupled with efficient solvation free energy calculations. *J. Chem. Theory Comput.* **12**, 5088–5099 (2016).
2. Giannozzi, P. *et al.* QUANTUM ESPRESSO: a modular and open-source software project for quantum simulations of materials. *J. Phys.: Condens. Matter* **21**, 395502 (2009).
3. Plimpton, S. Fast parallel algorithms for short-range molecular dynamics. *J. Comput. Phys.* **117**, 1–19 (1995).
4. Blöchl, P. E. Projector augmented-wave method. *Phys. Rev. B* **50**, 17953–17979 (1994).
5. Perdew, J. P., Burke, K. & Ernzerhof, M. Generalized gradient approximation made simple. *Phys. Rev. Lett.* **77**, 3865–3868 (1996).
6. Ringe, S. *et al.* Understanding cation effects in electrochemical CO_2 reduction. *Energy Environ. Sci.* **12**, 3001–3014 (2019).
7. Henkelman, G., Arnaldsson, A. & Jónsson, H. A fast and robust algorithm for Bader decomposition of charge density. *Comput. Mater. Sci.* **36**, 354–360 (2006).
8. Price, D. J. & Brooks, C. L. A modified TIP3P water potential for simulation with Ewald summation. *J. Chem. Phys.* **121**, 10096–10103 (2004).
9. Nosé, S. A unified formulation of the constant temperature molecular dynamics methods. *J. Chem. Phys.* **81**, 511–519 (1984).
10. Hoover, W. G. Canonical dynamics: equilibrium phase-space distributions. *Phys. Rev. A* **31**, 1695–1697 (1985).
11. Yeh, I.-C. & Berkowitz, M. L. Ewald summation for systems with slab geometry. *J. Chem. Phys.* **111**, 3155–3162 (1999).
12. Fyta, M. & Netz, R. R. Ionic force field optimization based on single-ion and ion-pair solvation properties: Going beyond standard mixing rules. *J. Chem. Phys.* **136**, 124103 (2012).
13. Jorgensen, W. L., Maxwell, D. S. & Tirado-Rives, J. Development and testing of the OPLS All-Atom force field on conformational energetics and properties of organic liquids. *J. Am. Chem. Soc.* **118**, 11225–11236 (1996).

14. Lee, K., Murray, É. D., Kong, L., Lundqvist, B. I. & Langreth, D. C. Higher-accuracy van der Waals density functional. *Phys. Rev. B* **82**, 081101 (2010).

15. Valiev, M. *et al.* NWChem: A comprehensive and scalable open-source solution for large scale molecular simulations. *Comput. Phys. Commun.* **181**, 1477–1489 (2010).

16. Rappoport, D. & Furche, F. Property-optimized Gaussian basis sets for molecular response calculations. *J. Chem. Phys.* **133**, 134105 (2010).

17. Shin, S.-J. *et al.* On the importance of the electric double layer structure in aqueous electrocatalysis. *Nat. Commun.* **13**, 174 (2022).

18. Gould, T. & Bučko, T. C_6 coefficients and dipole polarizabilities for all atoms and many ions in rows 1–6 of the periodic table. *J. Chem. Theory Comput.* **12**, 3603–3613 (2016).

19. Gould, T., Lebègue, S., Ángyán, J. G. & Bučko, T. A fractionally ionic approach to polarizability and van der Waals many-body dispersion calculations. *J. Chem. Theory Comput.* **12**, 5920–5930 (2016).

20. Nørskov, J. K. *et al.* Origin of the overpotential for oxygen reduction at a fuel-cell cathode. *J. Phys. Chem. B* **108**, 17886–17892 (2004).

21. Li, H. & Jensen, J. H. Partial Hessian vibrational analysis: the localization of the molecular vibrational energy and entropy. *Theor. Chem. Acc.* **107**, 211–219 (2002).

22. Calle-Vallejo, F. & Koper, M. T. M. Theoretical considerations on the electroreduction of CO to C2 species on Cu(100) electrodes. *Angew. Chem. Int. Ed.* **52**, 7282–7285 (2013).

23. Kim, Y. *et al.* Time-resolved observation of C–C coupling intermediates on Cu electrodes for selective electrochemical CO_2 reduction. *Energy Environ. Sci.* **13**, 4301–4311 (2020).

24. Dinh, C.-T. *et al.* CO_2 electroreduction to ethylene via hydroxide-mediated copper catalysis at an abrupt interface. *Science* **360**, 783–787 (2018).

25. Kortlever, R., Shen, J., Schouten, K. J. P., Calle-Vallejo, F. & Koper, M. T. M. Catalysts and reaction pathways for the electrochemical reduction of carbon dioxide. *J. Phys. Chem. Lett.* **6**, 4073–4082 (2015).

26. Bell, R. P. The theory of reactions involving proton transfers. *Proc. R. Soc. Lond. A* **154**, 414–429 (1936).

27. Evans, M. G. & Polanyi, M. On the introduction of thermodynamic variables into reaction kinetics. *Trans. Faraday Soc.* **33**, 448–452 (1936).

28. Bard, A. J. & Faulkner, L. R. Kinetics of electrode reactions. In *Electrochemical methods: fundamentals*

and applications. (John Wiley & Sons, Inc., 2001).

29. Newman, J. & Thomas-Alyea, K. E. Structure of the electric double layer. In *Electrochemical systems*. (John Wiley & Sons, Inc., 2004).
30. Trasatti, S. The 'absolute' electrode potential - the end of the story. *Electrochim. Acta* **35**, 269–271 (1990).
31. Trasatti, S. Work function, electronegativity, and electrochemical behaviour of metals. *J. Electroanal. Chem.* **33**, 351–378 (1971).
32. Xue, S. *et al.* Influence of alkali metal cations on the hydrogen evolution reaction activity of Pt, Ir, Au, and Ag electrodes in alkaline electrolytes. *ChemElectroChem* **5**, 2326–2329 (2018).
